# Rapid Thermal Shutdown of Deep‐Eutectic‐Polymer Electrolyte Enabling Overheating Self‐Protection of Lithium Metal Batteries

**DOI:** 10.1002/advs.202409628

**Published:** 2024-10-29

**Authors:** Zengqi Zhang, Gang Li, Xiaofan Du, Lang Huang, Guohong Kang, Jianjun Zhang, Zili Cui, Tao Liu, Ling Ni, Yongcheng Jin, Guanglei Cui

**Affiliations:** ^1^ School of Materials Science and Engineering Ocean University of China Qingdao 266100 P. R. China; ^2^ Qingdao Industrial Energy Storage Research Institute Qingdao Institute of Bioenergy and Bioprocess Technology Chinese Academy of Sciences Qingdao 266101 P. R. China; ^3^ Shandong Energy Institute Qingdao New Energy Shandong laboratory Qingdao 266101 P. R. China

**Keywords:** deep‐eutectic‐polymer electrolyte, lithium metal batteries, long cycle life, thermal safety, thermal shutdown

## Abstract

Safety concerns and uncontrollable dendrite growths have severely impeded the advancement of lithium‐metal batteries. Herein, a safe deep‐eutectic‐polymer electrolyte with built‐in thermal shutdown capability is proposed by utilizing hydrophobic association of methylcellulose within a novel deep‐eutectic‐solvent. Specifically, at elevated temperatures, methylcellulose chains aggregate to form dense polymer networks due to hydrophobic association and break the solvation structure equilibrium inside the deep‐eutectic system through encapsulating Li^+^ in polymer matrix, leading to quick solidification of the electrolyte. The solidified electrolyte obstructs Li^+^ transports and terminates electrochemical processes, protecting LMBs from unstoppable exothermic chain reactions. The accelerating rate calorimeter tests of 1 Ah pouch cells demonstrate that the as‐prepared electrolyte significantly improves the onset self‐heating temperature from 73 °C for conventional electrolytes to 172 °C and prolongs the thermal runaway waiting time more than 20 hours. More impressively, benefiting from its favorable electrochemical performance, this polymer electrolyte enables LiNi_0.8_Mn_0.1_Co_0.1_O_2_||Li batteries to retain 92% capacity over 200 cycles and LiFePO_4_||Li batteries to maintain 90% capacity after 500 cycles. This research paves a promising avenue for enhancing both the safety and electrochemical performance of high‐energy‐density LMBs.

## Graphical abstract

A deep‐eutectic‐polymer electrolyte with the thermal shutdown property is designed to improve thermal safety of lithium metal batteries. This electrolyte exhibits high conductivity and superior lithium compatibility at room temperature, endowing lithium metal batteries with excellent cycling performances. Meanwhile, it can solidify rapidly at elevated temperature, protecting the battery from disastrous thermal runaway.

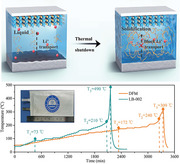

## 1 Introduction

**Figure 1:**
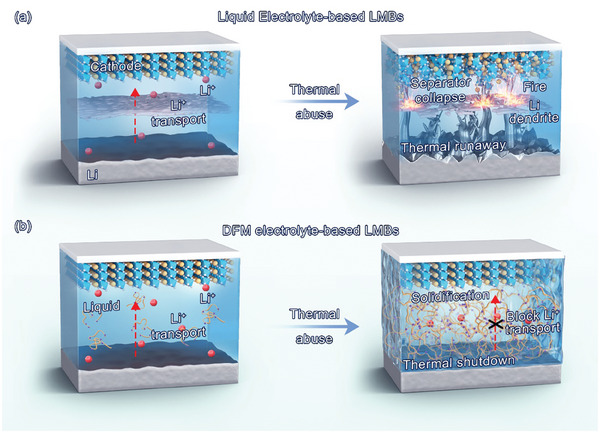
(a) Schematic illustration of the thermal runaway in LMBs employing commercially available electrolyte. (b) Schematic illustration of as‐prepared DFM electrolyte with thermal shutdown characteristics.

The booming developments in consumer electronics, electric vehicles, and energy storage systems, have revitalized worldwide efforts to exploit high‐energy‐density batteries.^[^ ^1^, ^2^, ^3^ ^]^ The lithium metal is a promising anode for designing batteries with an energy density exceeding 500 Wh kg^−1^ due to its lowest redox potential (−3.04 V vs SHE) and the highest specific capacity (3860 mAh g^−1^). However, severe safety hazards stemming from the inherent high chemical reactivity of metallic lithium and the growth of lithium dendrites hinder the practical application of lithium‐metal batteries (LMBs).^[^ ^4^, ^5^, ^6^ ^]^ Specifically, the sharp lithium dendrite can pierce through the separator and cause internal short‐circuit in LMBs, leading to serious thermal runaway. In addition, under abnormal abuse conditions, such as over‐charge/discharge, ultrahigh currents, high/low running temperatures, LMBs readily get overheated due to inevitable ohmic heating and electrochemical reaction heating.^[^ ^7^ ^]^ As the battery temperature increases to a certain degree, interfacial exothermic chain reactions are easily triggered, including solid electrolyte interface (SEI) decomposition and electrolyte/electrode reactions, resulting in further temperature rise in the batteries.^[^ ^8^, ^9^ ^]^ What's worse, as the temperature exceeds the melting point of polyolefin separators (165 °C for polypropylene and 140 °C for polyethylene),^[^ ^10^ ^]^ large‐area shrinkage and collapse of separators occur inevitably, leading to severe internal short‐circuit and catastrophic thermal runaway (**Figure** **1**a).^[^ ^11^, ^12^ ^]^ Therefore, it is priority to address safety hazards for the development of advanced LMBs.

Carbonate‐based electrolytes have long been a primary culprit responsible for the imperfect safety property of LMBs owing to their intrinsic flammability and undesirable thermo‐compatibility with electrodes at elevated temperatures. Over the past decade, substantial efforts have been directed to heighten safety characteristics of the electrolyte, such as development of solid‐state electrolyte, the addition of functional additives, and design of nonflammable electrolytes, etc.^[^ ^13^, ^14^ ^]^ The substitution of liquid electrolytes with inorganic solid‐state electrolytes holds high expectation to address the safety issues in LMBs attributing to the excellent thermal stability of solid electrolyte. Nonetheless, deficient solid‐solid interfaces between electrodes and electrolytes remain a great challenge for achieving satisfying electrochemical performance.^[^ ^15^ ^]^ Incorporating flame retardant additives, such as triethyl phosphate, tributylphosphate, and triphenyl phosphate, into liquid electrolytes is another feasible strategy to reduce burning risks due to their free radicals scavenging mechanisms.^[^ ^16^ ^]^ Yet, their inferior compatibility with anodes could potentially cause lithium dendrites growth, deteriorating the coulombic efficiency (CE) and cycling performance of the battery.^[^ ^17^, ^18^, ^19^ ^]^ Recently, polymers with thermal responsive function are incorporated into liquid electrolyte to build smart batteries, for example, in situ polymerizations are designed inside electrolyte to shutdown batteries at elevated temperatures.^[^ ^20^ ^]^ However, introducing thermal responsive polymer into traditional carbonate solvents still presents high flammability risks owing to flammability of the latter. Moreover, the responding times of most reported thermal shutdown electrolyte is too long to prevent the thermal runaway.^[^ ^21^ ^]^

Deep‐eutectic‐solvent (DES), as an emerging kind of eutectic mixture, exhibits high thermal stability, nonflammability, and excellent ionic conductivity, serving as an promising alternative candidate to improve the safety property of LMBs.^[^ ^22^, ^23^, ^24^ ^]^ More interestingly, the internal solvation structure is the key to facilitate the transition of DES system from solid to liquid state, which means manipulating intermolecular interactions could also change the DES from liquid into solid state, opening a new door for developing quick phase‐change electrolyte systems. Therefore, synergistically combining DES with thermal‐responsive polymers holds great potential to design an electrolyte that can rapidly shut down battery operation under elevated temperature. Based on the aforementioned analysis, herein, a novel polymer electrolyte (DFM) with a rapid thermal shutdown function is proposed by utilizing hydrophobic association of methylcellulose (MC) and N,N,N’,N’‐tetramethylsulfonamide‐based DES (Figure S1, Supporting Information). Specifically, at elevated temperatures, MC chains rapidly aggregate to form dense polymer networks owing to hydrophobic association, altering solvation structure of the deep‐eutectic system by trapping lithium ions into polymer matrixes. This consequently leads to the quick solidification of the polymer electrolyte (Figure S2, Supporting Information), which obstructs Li^+^ transportation and terminates the electrochemical process of the LMBs, relieving the thermal runaway (Figure 1b). Compared with the previously reported thermo‐responsive electrolytes, this polymer electrolyte terminates the electrochemical process in a shorter time at elevated temperature due to the synergistic effect from both MC and DES, significantly suppressing heat release in the initial stage of thermal runaway. Accelerating rate calorimeter (ARC) tests of 1 Ah pouch cells confirm that, compared with commercially available electrolytes, this polymer electrolyte improves the self‐heating temperature of the LMBs from 73 to 172 °C, and prolongs the slow self‐heating time by more than 20 h, greatly alleviating the thermal runaway behavior. Meanwhile, the nonflammability of DES‐based electrolyte remarkably reduces the total heat release of pouch cells during thermal runaway. More impressively, benefitting from excellent electrochemical properties of the as‐prepared DFM electrolyte, the LiNi_0.8_Mn_0.1_Co_0.1_O_2_(NCM811)||DFM||Li batteries display superior capacity retention of 92% after 200 cycles at 0.5 C rate, and LiFePO_4_||DFM||Li batteries keep 90% of capacity after 500 cycles at 1 C rate. This deep‐eutectic‐polymer electrolyte simultaneously promoting safety characteristics and electrochemical performances of LMBs delivers great inspiration on developing next‐generation safe and high energy density batteries.

## 2 Results and Discussion

### 2.1 Structure Characterization and Physicochemical Properties of the DFM Electrolyte

**Figure 2:**
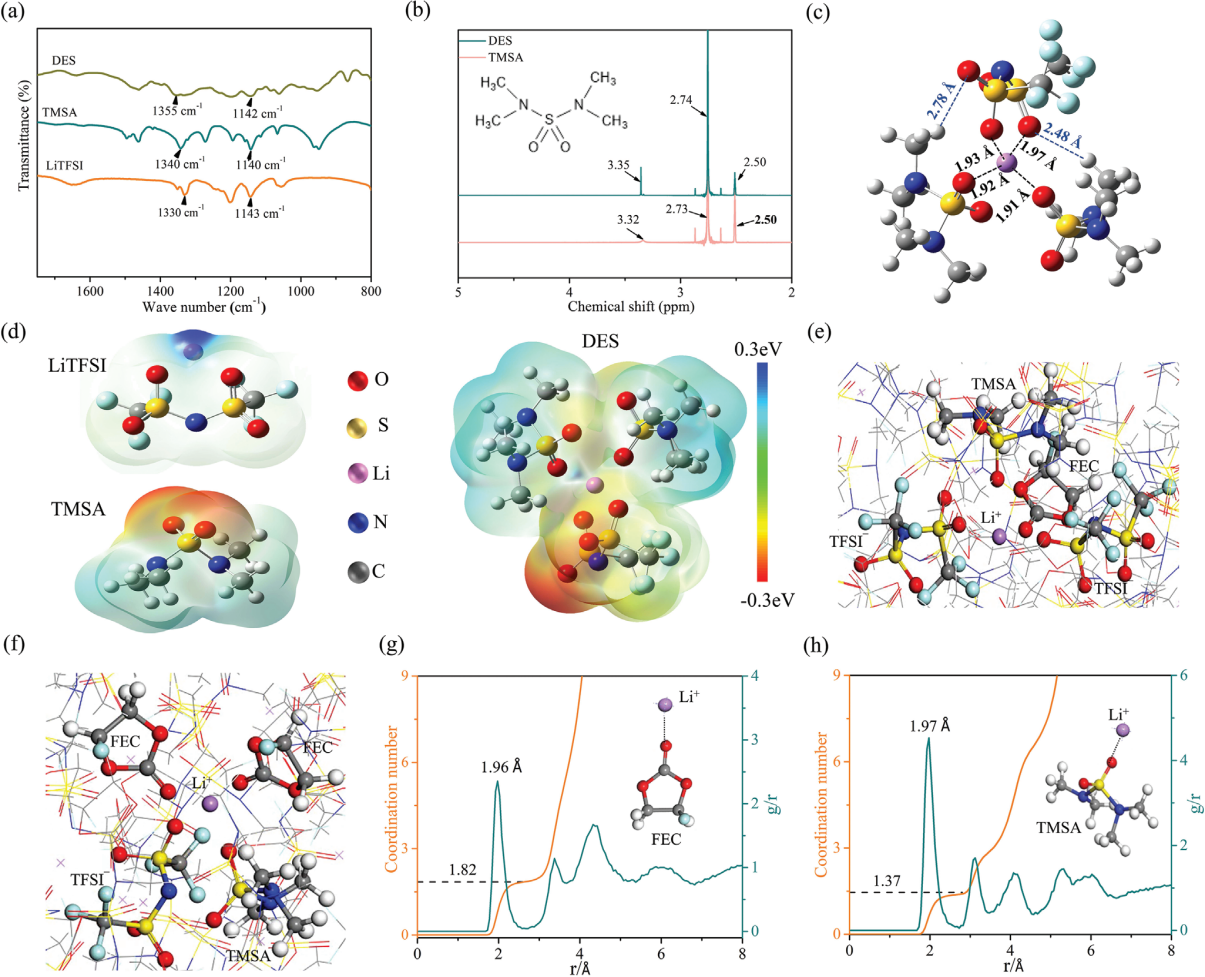
Electrolyte characterization: (a) Fourier transform infrared spectra (FTIR) and (b) ^1^H nuclear magnetic resonance (^1^H NMR) spectra of TMSA, LiTFSI, and DES. (c) Optimizing molecular configuration of DES. (d) Electrostatic potential (ESP) mapped on total electron density of LiTFSI, TMAS, and DES. (e,f) Representative Li^+^ complex structures (magnified local solvation structure during MD simulations) in DFM. Radial distribution function (RDF) of (g) Li‐FEC and (h) Li‐TMSA.

To prepare the electrolyte, N,N,N“,N”−tetramethylsulfonamide (TMSA) was blended with LiTFSI (molar ratio, 2:1) to form DES, and then FEC and MC were added into DES to obtain the deep‐eutectic‐polymer electrolyte (shorted as DFM). FTIR and ^1^H NMR spectra were employed to analyze the molecular interactions in the DES (**Figure** **2**a,b). In FTIR spectra, the asymmetric stretching vibration of S≐O for TMSA and LiTFSI is found at 1340 and 1330 cm^−1^, respectively. In the DES, the S≐O of TMSA coordinates with Li^+^ spontaneously, reducing the electron density of S≐O, thereby the peak corresponding asymmetric stretching vibration of S≐O (TMSA) shifts to 1320 cm^−1^. In contrast, the strong interactions between Li^+^ and TMSA facilitate Li^+^ dissociation from TFSI^−^, resulting in a blue shift of S≐O (TFSI^−^) peak to 1355 cm^−1^.^[^ ^25^ ^]^ Based on the above results, coordination bonds of Li‐TMSA are dominating intermolecular forces inducing DES formation. ^1^H NMR of DES displays singlets at 2.50, 2.74, and 3.35 ppm, which corresponds to D of dimethyl sulfoxide‐D6, H of −CH_3_ (TMSA), and H of water molecule, respectively (Figure 2b).^[^ ^26^, ^27^ ^]^

Theoretical calculation gives further information to reveal the formation mechanism of DES. Electronic static potentials (ESPs) deliver vivid evidence to find potential interaction sites between molecules (Figure 2d). The dark blue around Li^+^ in LiTFSI molecules suggests high electron deficiency, while the bright red indicates the electron‐rich near O atoms of TMSA. It can be found that the blue around Li^+^ of LiTFSI and the red near O of TMSA fade obviously in the molecular configuration of DES, indicating that O (TMAS) forms coordination bond with Li^+^ in DES. The results suggest that the TMSA, as competitive ligand, coordinates with Li^+^ and weakens interaction between Li^+^ and TFSI^−^ to some extent, which facilitates Li^+^ dissociation from TFSI^−^.^[^ ^27^ ^]^ Figure 2c shows optimizing state of molecular configuration of DES, in which Li^+^ coordinates with one TFSI^−^ and two TMSA molecules. The length between O of TMSA and Li^+^ was 1.92 Å, while the distance between Li^+^ and O of TFSI^−^ is 1.97 Å, which confirms that the Li^+^‐TMSA interaction may be as strong as Li^+^‐TFSI^−^. Furthermore, two H atoms of TMSA are found in positions of 2.48 and 2.78 Å away from O of TFSI^−^, indicating that TMSA probably forms weak hydrogen bond with TFIS^−^. The theoretical calculation verifies that strong coordination bonds of Li‐TMSA and interactions between TMSA and TFSI^−^ are dominating intermolecular forces for DES.

DFM is obtained by adding FEC and the thermo‐responsive polymer of MC into DES. Generally, FEC facilitates the generation of stable solid electrolyte interfaces on Li surface to optimize the charge transport over the electrolyte/Li interphase, while the thermally responsive MC enables electrolytes to achieve thermal shutdown at elevated temperature.^[^ ^28^, ^29^ ^]^ In the spectra of FEC, multiplets at 4.70 ppm correspond to ‐CH_2_‐, and peaks at 6.52 and 6.64 ppm are assigned to ‐CHF‐. It can be found that the characteristic peaks of H (FEC) show negligible chemical shift after forming DFM electrolyte, indicating that no chemical reactions take place between FEC and DES (Figures S3 and S4, Supporting Information).^[^ ^30^ ^]^ To vividly reveal the solvation structure of Li^+^ in DFM, theoretical calculation of density functional theory (DFT) and molecular dynamics (MD) are employed. The detailed MD results reveal that two kinds of primary coordination shell of Li^+^ can be found in DFM, including Li^+^‐2FEC‐TMSA‐TFSI^−^ and Li^+^‐FEC‐TMSA‐2TFSI^−^ (Figure S5, Supporting Information; Figure 2e,f). The existence of FEC in primary solvation structure is beneficial for forming stable SEI at the surface of Li metal. The radial distribution function (RDF) shows sharp peaks of both FEC and TMSA molecules at 1.9 and 1.97 Å away from the Li^+^ (Figure 2g,h), which manifests that FEC and TMSA participate in the Li^+^ primary coordination shell.

**Figure 3:**
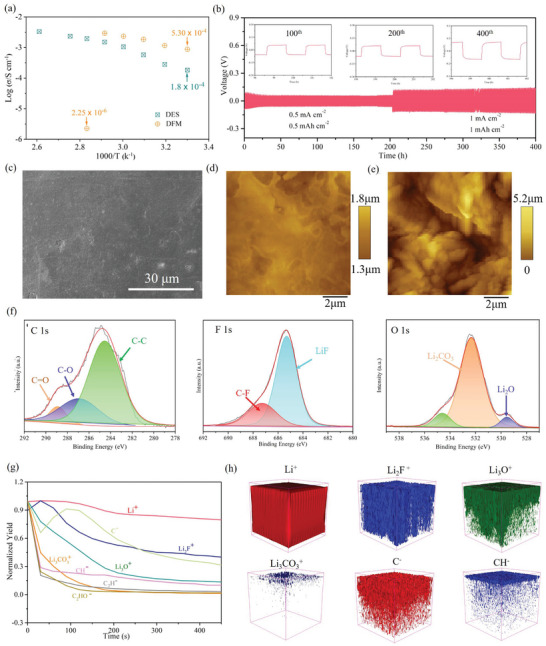
The properties of as‐prepared electrolyte and interfacial characterization of cycled Li anode. (a) Ionic conductivities of DES and DFM electrolytes as a function of temperature. (b) Voltage profiles of Li||DFM||Li symmetric cells at current density 0.5 and 1 mA cm^−2^ with constant capacity of 0.5 and 1 mAh cm^−2^, respectively. (c) Typical SEM and (d) AFM images of Li electrodes after 100 plating/stripping cycles in Li||DFM||Li symmetric cells. (e) AFM images of Li electrodes after 100 plating/stripping cycles in Li||LB‐002||Li symmetric cells. (f) XPS spectra (C1s, O1s, and F1s) of Li surface after cycling in Li||DFM||Li symmetric cells. (g) TOF–SIMS depth profiles of several typical second ion fragments on the cycled Li electrodes of DFM cell. (h) Reconstructed 3D spatial fragment distribution of selected species.

Thermal gravimetric analyzer (TGA) is employed to investigate the thermal stability of the prepared electrolyte. Given its low vapor pressure, DFM displays a low weight loss (2.77%) until 150 °C, suggesting that neither volatilization nor degradation takes place in DFM electrolyte below 150 °C (Figure S6, Supporting Information). As comparison, the commercially available electrolyte (LB‐002) loses 4.79% of original weight at room temperature because of quick evaporation of carbonate solvents, and presents a weight loss of 51.77% at 150 °C. Furthermore, the flammability of DFM is investigated through a combustion test. As shown in Figure S7 (Supporting Information), the DFM cannot be ignited, while LB‐002 burns violently when it is close to flame. The high thermal stability and excellent nonflammability of DFM would drastically improve the safety of high‐energy‐density LMBs. The dependence of the ionic conductivity of electrolytes on temperature is evaluated by EIS (**Figure** **3**a). Because of its high viscosity (517 mPa s, Figure S8, Supporting Information), the DES displays a relatively low ionic conductivity of 1.8  × 10^−4^ S cm^−1^ at room temperature. Compared with DES, DFM exhibits a lower viscosity of 357 mPa s owing to the addition of FEC, and provides a higher ionic conductivity of 5.30  × 10^−4^ S cm^−1^ at room temperature. In the temperature range of 30–70 °C, the ionic conductivity of DFM increase with the rise of temperature. However, when temperature exceeds 80 °C, DFM solidifies rapidly, which blocks Li^+^ transport. The ionic conductivity of DFM after heating at 80 °C for 2 min is 2.25  × 10^−6^ S cm^−1^ (Figure S9, Supporting Information). In addition, the Li^+^ transference number (*t_Li+_*) of DFM is 0.413 at room temperature, and drastically reduces to 0.147 after electrolyte solidifying (Figure S10, Supporting Information). The obvious diminutions of ionic conductivity and Li^+^ transference number under thermal stimulation are the basis for overheating self‐protection of DFM. The mechanism for the above phenomenon can be explained as follows: the MC molecules are hydrated as hydrophobic segments and surrounded by solvent molecules through interaction between MC and DES at room temperature, forming an enclosed cagelike structure. Thereby the MC dispreases in DES as randomly coiled and isolated chains at room temperature, in which the Li^+^ can migrate freely.^[^ ^31^ ^]^ However, as the temperature exceeds 80 °C, the MC structures are gradually distorted due to weakened solvent‐MC interactions. Meanwhile, the Li^+^‐MC interaction and hydrophobic interactions between −OCH_3_ of MC becomes stronger.^[^ ^32^ ^]^ As a result, the MC chains are rapidly distorted and tangled to form hydrophobic aggregates and eventually creates infinite crosslinked networks, which causes the quick solidification of electrolyte.^[^ ^33^ ^]^ After electrolyte solidifying, most of Li^+^ are trapped in gel networks because of the enhanced electronic interaction between MC chains and Li^+^. This changes solvation structure equilibrium inside the DES, and increases viscosity of the DES, facilitating electrolyte solidification. Therefore, both of the ionic conductivity and *t_Li+_* decrease significantly after electrolyte solidification at 80 °C. The data of liner sweep voltammetry (LSV) for Li─SS cells demonstrate that DES shows stable potential of 4.43 V, while DFM delivers stable potential of 4.32 V probably due to the decomposition of FEC (Figure S11, Supporting Information).

To reveal the compatibility of electrolyte with Li metal, the plating/striping cycles of Li||Li symmetric cells are conducted at currents of 0.5 and 1 mA cm^−2^ with constant capacities of 0.5 and 1 mAh cm^−2^, respectively (Figure S12, Supporting Information; Figure 3b). The Li||LB‐002||Li cell displays a stable overpotential of 66 mV at current density of 0.5 mA cm^−2^. However, when the current density increases to 1 mAh cm^−2^, the overpotential of cells distinctly increases to 0.5 V due to inferior SEI. Obviously, Li||DES||Li shows asymmetric plating/striping voltage files with an overpotential of 0.1 V in the initial cycles (Figure S12, Supporting Information). Although the overpotential of Li||DES||Li cells decreases to 20 mV after 10 cycles, it distinctly increases to 0.5 V at a current density of 1 mA cm^−2^. The reason is that DES cannot form protective SEI on the Li surface, thereby parasitic reactions between the DES and Li metal take place continuously during Li^+^ plating/striping cycle. In contrast, the symmetric cells of Li||DFM||Li deliver reduced and stable voltage hysteresis during plating/striping process. A conclusion can be drawn that DFM generates stable SEI on Li surface, facilitating uniform deposition of Li^+^ and inhibiting the occurrence of parasitic reaction. SEM and AFM (Atomic Force Microscope) are conducted to observe surface morphologies of Li electrodes after 100 plating/stripping cycles in symmetric cells at a current density of 0.5 mA cm^−2^ (0.5 mAh cm^−2^). The cycled Li electrode of Li||DFM||Li cells displays a smooth surface with few cracks and dendrites, suggesting homogeneous Li^+^ disposition (Figure 3c,d). In contrast, the Li anode obtained from cycled Li||LB‐002||Li cell displays an obviously porous and loose surface due to uneven Li disposition and parasitic interface reactions (Figure S13a, Supporting Information; Figure 3e). Similarly, apparent particles and cracks can be found on the surface of Li electrodes harvesting from the cycled Li||DES||Li cell, which verifies that DES hardly protects Li from parasitic interface reactions owing to inferior SEI (Figure S13b, Supporting Information). The results certify that DFM electrolytes greatly mitigate the parasitic reaction and effectively induce uniform lithium stripping/deposition through generation of the protective SEI on the Li metal surface, preventing the lithium dendrites from piercing separator and causing internal short‐circuit in the LMBs. The energy‐dispersive X−ray spectrometer (EDS) test displays that elements of F, C, and O are detected on the cycled Li surfaces of Li||DFM||Li cells, which reveals that the surface layer on Li metal mainly includes F, C, and O containing species (Figure S14, Supporting Information).

To analyze the chemical composition of SEI, X‐ray photoelectron spectroscopy (XPS) is further employed. Three fitting peaks can be seen at 284.8, 286.8, and 289.6 eV in C 1s spectra of Li (Figure 3f), which are attributed to characteristic signals of C─C in organic compounds, LiCOOR and Li_2_CO_3_, respectively. The characteristic peak on the F1s spectra at 685.3 eV is assigned to LiF,^[^ ^34^ ^]^ while two fitting peaks at 529.0 and 533.3 eV on the O 1s spectra corresponds to Li_2_O and Li_2_CO_3_, respectively (Figure 3f).^[^ ^35^ ^]^ The XPS results confirm that SEI is mainly composed of inorganic and organic species, including LiF, Li_2_CO_3_, Li_2_O, and LiCOOR. Compared with SEI formed in DES electrolyte (Figure S15, Supporting Information), that formed in DFM show a higher content of inorganic LiF and Li_2_CO_3_, which could improve the thermal stability of SEI. Time‐of‐flight secondary ion mass spectrometry (TOF‐SIMS) is an effective method to investigate the interfacial composition on the Li metal (Figure 3g,h). For the Li electrodes from Li||DFM||Li cells, several kinds of fragments, such as Li^+^, Li_2_F^+^, Li_3_O^+^, Li_2_CO_3_^+^, and anion of C^−^, CH^−^ are detected under positive and negative model, respectively, further confirming the existence of LiF, Li_2_O, Li_2_CO_3_ and organic specific (−COOR) in SEI.^[^ ^36^ ^]^ It can be found that the intensities of most fragments reduce with increase of sputtering time, and keep relatively low content after 400 s, indicating that the interfacial composition distributes in depths within sputtering time 0–400 s. Given that the sputtering depth is 10 nm in 60 s, the thickness of SEI is ≈70 nm. The organic fragments, such as C_2_HO^−^, CH^−^, and C_2_H^−^, decrease continuously with the deepening of sputtering, manifesting that the organic compounds are mainly located in the outer layer of SEI.^[^ ^37^ ^]^ Figure 3h is reconstructed 3D spatial fragment distributions, which gives a direct visual picture to investigate distribution of fragments in each layer of SEI. Obviously, inorganic fragments of Li_2_F^+^ mainly aggregates in the inner layer of SEI (close to Li metal), and it regulates the Li^+^ deposition at the electrode and relieves the generation of Li dendrites. Whereas, the organic fragments of CH^−^ are observed in the outer layer of SEI, increasing the elastic modulus of SEI.^[^ ^38^ ^]^

### 2.2 Electrochemical Performance of LMBs Employing DFM Electrolyte

**Figure 4:**
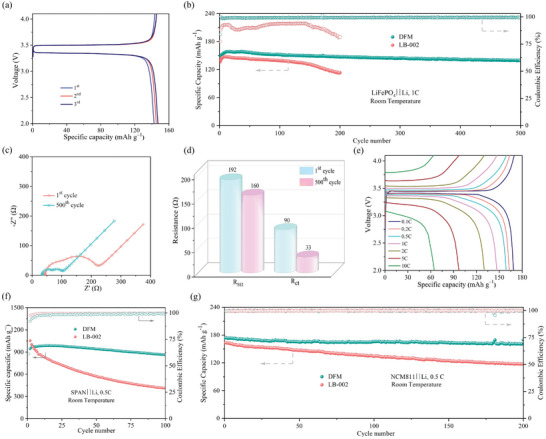
Electrochemical performances of LMBs: (a) Galvanostatic charge/discharge profiles and (b) Cycling performances of the LiFePO_4_‐based LMBs (LiFePO_4_||Li) at room temperature and 1 C. (c) EIS and (d) *R_SEI_* and *R_ct_* of LiFePO_4_||DFM||Li employing the DFM electrolytes after the 1st and 500th cycle. (e) Galvanostatic charge/discharge profiles of LiFePO_4_||DFM||Li at room temperature and different current densities (0.1, 0.2, 0.5, 1, 2, 5, and 10 C). (f) Cycling performances of sulfurized polyacrylonitrile‐based LMBs (SPAN||Li) and (g) NCM811‐based LMBs (NCM811||Li).

LiFePO_4_‐based batteries are assembled to assess the electrochemical performance of electrolytes. The cyclic voltammogram (CV) curves of LiFePO_4_||DFM||Li batteries display a pair of typical peaks corresponding to oxidation and reduction of LiFePO_4_, which confirms that only redox reactions of LiFePO_4_ take place (Figure S16, Supporting Information). **Figure** **4**a,b shows that the initial specific capacity of LiFePO_4_||DFM||Li is 143 mAh g^−1^ at 1 C (room temperature), and remains 90% of the initial capacity after 500 cycles. As comparison, the LB‐002 battery exhibits inferior performance with a reversible capacity of 147 mAh g^−1^ in the first cycle, and remains 68.1% after 200 cycles. The coulombic efficiency of DFM battery in the first cycle is 88% due to the formation of SEI, while it rapidly increases to 100% in the second cycle and remains stable until 500 cycles. However, because parasitic reactions take place continuously at interfaces of electrolyte/Li, LB‐002 battery shows unsatisfactory coulombic efficiency during long‐term cycle. The EIS of batteries was measured after the 1st and 500th cycles (Figure 4c,d). Two well‐defined semicircles at high and middle frequencies correspond to the diffusion impedance of Li^+^ transport through SEI (*R_SEI_*) and interfacial charge transfer resistance (*R_ct_*), respectively, and the sloping line at low frequency is attributed to the Warburg impedance (*R_w_*) representing the diffusion rate of Li^+^ in the bulk of LiFePO_4_. After 1st cycle, the *R_SEI_* and *R_ct_* of DFM battery are 191 and 159 Ω, and they reduce to 89 and 32 Ω after 500 cycles, respectively. The results reveal that stable SEI generating in DFM facilitates Li^+^ transport through electrolyte/Li interfaces.

The performance of LiFePO_4_||DFM||Li under different current densities is shown in Figure 4e. The initial discharge capacity of LiFePO_4_||DFM||Li batteries is 167 mAh g^−1^ at 0.1 C, which is close to theoretically specific capacity of LiFePO_4_. With the rise of current density, the polarizations of voltage profiles increase owing to limitation of reaction kinetics, reducing discharge capacity to some extends. The LiFePO_4_||DFM||Li battery delivers specific capacities of 162, 158, 146, 130, 97, and 65 mAh g^−1^ at 0.2, 0.5, 1, 2, 5, and 10 C, respectively. As comparison, the LiFePO_4_||LB‐002||Li battery delivers specific capacities of 140, 136, 135, 130, 115, 78, and 43 mAh g^−1^ at 0.1, 0.2, 0.5, 1, 2, 5, and 10 C (Figure S17, Supporting Information). It is worth noting that LiFePO_4_||DFM||Li recovers almost 100% of initial capacity when the current density returns to 0.1 C from 10 C, while LiFePO_4_||LB‐002||Li recovers only 83% of the initial capacity under the same circumstance. Compared with the LiFePO_4_||LB‐002||Li battery, the higher specific capacity and improved reversibility of LiFePO_4_||DFM||Li batteries derive from optimization on charge transport over electrolyte/Li interface. To verify the universal applicability of DFM electrolyte in other battery systems, LMBs with cathodes of sulfurized polyacrylonitrile (SPAN) are assembled and tested (Figure S18, Supporting Information). The first discharge process corresponds to the irreversible conversion of the original SPAN with unique Li^+^ storage mechanism, delivering a specific capacity of 1600 mAh g^−1^. In subsequent cycles, SPAN||DFM||Li displays the reversible charge/discharge processes with a specific capacity of 945 mAh g^−1^, and remains capacity of 880 mAh g^−1^ after 100 cycles at a current density of 0.5 C, which is better than that using LB‐002 (Figure 4f). Additionally, the cycling performance of LiNi_0.8_Co_0.1_Mn_0.1_O_2_‐based LMBs (NCM811||Li) is assessed. After activation at current density of 0.1 C, the NCM811||Li battery employing DFM delivers a discharge capacity of 175 mAh g^−1^, and keeps 161 mAh g^−1^ after 200 cycles. In comparison, the NCM811||Li batery with LB‐002 electrolyte displays inferior cycling performance with an initial discharge capacity of 163 mAh g^−1^ and a capacity retention of 118 mAh g^−1^ after 200 cycles. The results confirm that the as‐prepared DFM electrolyte endows LMBs with improved cycle performances due to its excellent compatibility with Li anode.

### 2.3 Overheating Self‐Protection of LMBs using DFM Electrolyte

**Figure 5:**
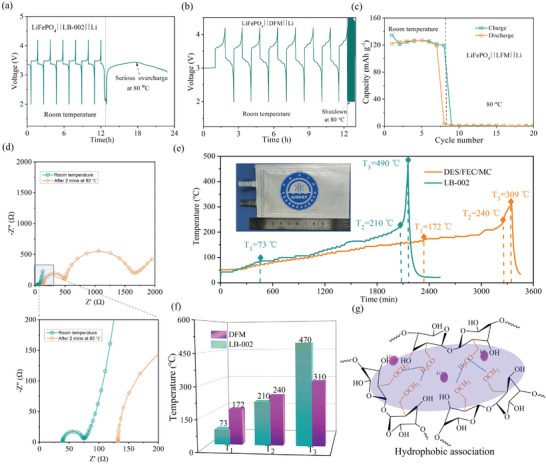
Thermal performance of batteries. Voltage profiles of LiFePO_4_||Li cells using (a) commercially available electrolyte (LB‐002) and (b) as‐prepared DFM electrolytes at room temperature and 80 °C (1 C). (c) Charge/discharge specific capacity of the LiFePO_4_||DFM||Li batteries at room temperature and 80 °C (1 C). (d) Electrochemical impedance spectroscopy of LiFePO_4_||DFM||Li battery at room temperature and at 80 °C. (e) Accelerating rate calorimeter (ARC) test results of LiFePO_4_ punch cells with the electrolytes of LB‐002 and DFM. (f) Comparisons of ARC results (*T_1_*, *T_2_*, *T_3_*) for LiFePO_4_||Li pouch cells with different electrolytes. (g) Schematic illustration of hydrophobic association of MC at the elevated temperature.

LiFePO_4_ cells with DFM and LB‐002 are assembled and tested to evaluate their thermal behaviors. At 80 °C, the LiFePO_4_||LB‐002||Li battery undergoes serious overcharge with a specific capacity of 1600 mAh g^−1^ which is more than nine times of theoretically specific capacity of LiFePO_4_ (**Figure** **5**a; Figure S19, Supporting Information). The continuous overcharge would generate tremendous heat in the battery and trigger disastrous fire or explosion easily in large‐format batteries. In contrast, the DFM battery shows voltage profiles with an extremely high polarization and a negligible discharge capacity when the temperature sharply rises to 80 °C, indicating the termination of charge/discharge process (Figure 5b,c; Figure S20, Supporting Information). The EIS results indicate that the interfacial impedance of LiFePO_4_||DFM||Li significantly increases to 1700 Ω after electrolyte solidifying at 80 °C (Figure 5d). To reveal the reason for the significant increase of interface resistance, morphologies of the cathode surface are observed before and after electrolyte solidifying. As shown in Figure S21 (Supporting Information), a layer of polymer matrixes covers on the surfaces of cathode particles after electrolyte solidification, and it significantly blocks Li^+^ transportation over electrolyte/Li interfaces, leading to remarkable increase of interfacial resistance. The results demonstrate that the DFM can rapidly terminate Li^+^ transport at elevated temperature which is important to suppress rapid development of thermal runaway.

The temperature evolution in 1 Ah LiFePO_4_ pouch cells at 100% SOC (state of charge) was studied through ARC test. Three critical temperatures are marked on curves to analyze the thermal behaviors of cells (Figure 5e), including the onset temperature of self‐heating process (*T_1_*), thermal runaway temperature (*T_2_*), and maximum temperature (*T_3_*).^[^ ^39^, ^40^ ^]^ The self‐heating of LiFePO_4_||LB‐002||Li cell starts at 73 °C (*T_1_*), while the DFM electrolyte significantly increases *T_1_* of batteries to 172 °C. Commonly, *T_1_* is related to decomposition of SEI, the lower *T_1_* of LiFePO_4_||LB‐002||Li cell reveals that its SEI is inferior thermal stability and easily degrades as the temperature increase probably due to low inorganic species in SEI. Subsequently, the exothermic reactions between electrolyte and electrolyte take place due to decomposition of SEI, accelerating the temperature increase in the LMBs. As the temperature continuously rises, the separator collapses unavoidably in LB‐002 cell, as a result, internal short‐circuit occurs accompanying with the large current and the tremendous heat release. In the case of DFM‐based cells, the highly stable SEI eliminates the exothermic reaction that commonly occurs in carbonate solvents when the temperature increase, and reduces the produce of flammable gases, thereby retarding the self‐heating process. When the temperature exceeds 80 °C, the hydrophobic interactions of MC substituent (−OCH_3_) are enhanced (Figures S22 and S23, Supporting Information), thereby the MC chains assemble to form dense polymer networks (Figure 5g), leading to the electrolyte solidification. The polymer networks trapped the majority of Li^+^ into polymer matrixes due to the electronic interaction between MC chains and Li^+^. This alters the solvation structure of the deep‐eutectic‐solvent and dramatically reduces mobility of DES, facilitating the solidification process. Thereby, the synergistic effects of MC hydrophobic association and solvation structural changes of DES lead to the rapid solidification of the electrolyte at elevated temperature, which cuts off the Li^+^ transport completely and terminates battery operation. Additionally, the solidified electrolyte effectively prevents the direct contact of electrodes after the separator collapses, eliminating internal short‐circuit and alleviating heat release. Consequently, the automatic shutdown property of DFM protects cells from serious overcharge and relieves heat release under abuse condition, achieving overheating self‐protection in the batteries. Compared with LB‐002, DFM electrolyte also promotes the *T_2_* from 210 to 240 °C (Figure 5f), and prolongs the starting time of exponential heat‐generation from 2030 to 3340 min. The results confirm that the DFM electrolyte alleviates thermal runaway through automatically thermal response, unfolding a promising approach to building safety‐enhanced batteries. Furthermore, due to the excellent nonflammability, the DFM electrolyte significantly reduces the total heat accumulation, leading to a lower *T_3_* in LiFePO_4_||DFM||Li (310 °C) than that in LiFePO_4_||LB‐002||Li (470 °C). It can be found that the DFM effectively enhances the thermal compatibility between electrolyte and electrodes and delays the starting time of thermal runaway by spontaneous gelation. As a result, the DFM electrolyte alleviates thermal runaway through automatic thermal response, unfolding a promising approach to building safety‐enhanced batteries.

## 3 Conclusion

In summary, a safe deep‐eutectic‐polymer electrolyte of DFM with thermal shutdown functions and outstanding noninflammability is designed to achieve overheating self‐protection in LMBs at the elevated temperature. At room temperature, DFM keeps liquid state, and Li^+^ migrates freely between electrodes, thereby it displays a favorable conductivity of 5.30  × 10^−4^ S cm^−1^ and superior compatibility with Li metal. Consequently, the DFM‐based LMBs show excellent electrochemical performances. The NCM811||DFM||Li batteries deliver a specific capacity of 175 mAh g^−1^ at 0.5 C and keep 161 mAh g^−1^ after 200 cycles, and the LiFePO_4_||DFM||Li batteries deliver a capacity of 143 mAh g^−1^ at 1 C and show a capacity retention of 90% after 500 cycles. Interestingly, when the temperature exceeds 80 °C, the synergistic effects of MC hydrophobic association and solvation structural changes of DES result in rapid electrolyte solidification within 2 min. The solidified electrolytes terminate the electrochemical process through cutting off Li^+^ transportation between electrodes, which eliminates the thermal runaway in LMBs. The ARC tests of 1 Ah pouch cells demonstrate that the as‐prepared DFM electrolyte improves *T_1_* of pouch cells from 73 °C for conventional electrolyte to 172 °C and extends the slow self‐heating times by more than 20 hours. Furthermore, the excellent nonflammability of DFM diminishes total heat release, reducing the *T_3_* of the pouch cell. This work sheds light on the development of high‐safety and high‐energy‐density LMBs.

## Conflict of Interest

The authors declare no conflict of interest.

## Supporting information



Supporting Information

## Data Availability

Research data are not shared.
