# Supplementary material for: Rapid Thermal Shutdown of Deep‐Eutectic‐Polymer Electrolyte Enabling Overheating Self‐Protection of Lithium Metal Batteries
Source: Adv Sci (Weinh). 2024 Oct 29;11(48):2409628. doi: 10.1002/advs.202409628 (PMC11672307; doi:10.1002/advs.202409628)
Supplement: Supplementary file 1 — Supporting Information [file ADVS-11-2409628-s001.docx]

Supporting Information

Rapid thermal shutdown of deep-eutectic-polymer electrolyte enabling overheating self-protection of lithium metal batterie

Zengqi Zhang,^╂^ Gang Li,^╂^ Xiaofan Du,^╂^ Lang Huang,* Guohong Kang, Jianjun Zhang, Zili Cui, Tao Liu*, Ling Ni, Yongcheng Jin,* Guanglei Cui*

**I. Supporting Text**

**1. Experimental Section**

*1.1 Materials and instruments*:

N,N,N',N'−tetramethylsulfonamide (TMSA) was purchased from Alfa Aesar-China, and was dried overnight at 60 °C before using. Anhydrous grade bistrifluoromethanesulfonimide lithium (LiTFSI). Fluoroethylene carbonate (FEC), and methylcellulose (MC) were purchased from Sigma−Aldrich, the content of -CH_3_ is 27.5-31.5%. Other reagents were purchased from Aladdin Reagent. All materials were stored in an argon−filled glove box with oxygen and moisture content less than 0.1 ppm.

Fourier transform infrared spectra (FTIR) were conducted by Nicolet 6700 at room temperature; Nuclear magnetic resonance (NMR) analysis was performed on a Bruker AVANCE III 600 MHz with dimethyl sulfoxide (DMSO) as solvent. The surface morphologies of lithium were observed by scanning electrolytic microscopy (SEM) using a field emission scanning electron microscope (Hitachi S−4800). DSC214 was used to analyze thermal stability of electrolyte in the temperature range of 25 °C to 350°C with a ramp rate of 5 °C/min under nitrogen protection. X−ray photoelectron spectroscopy (PHI 5000 Versaprobe III) and time of flight secondary ion mass spectrometry (PHI nano TOF II Time−of−Flight SIMS) were employed to analyze interfacial composition.

*1.2 Electrolyte Preparation:*

TMSA and LiTFSI were blended with molar ratio of 2:1. The mixture was stirred overnight at room temperature to obtain solution of DES. 10% of FEC was added into DES by stirring, furthermore, 4.0% MC was introduced into electrolyte to generate DFM. Commercial electrolyte of LB-002, consisting of 1M LiPF_6_ in EC/DMC/EMC, was chose as control electrolyte.

*1.3 Electrochemical Characterization*:

Electrochemical impedance spectroscopy (EIS) was performed on a VMP−300 workstation in the frequency range from 7 MHz to 100 mHz with a perturbation amplitude of ±10 mV. The ionic conductivities were measured by using two stainless steel (SS) blocking electrodes over a frequency range of 7 MHz to 100 mHz with 10 mV. The ionic conductivity was calculated by Equation (1)

$\sigma=\frac{l}{(R_{b}*S)}$ Equation (1)

Where σ is ionic conductivity (S cm^−1^), *R_b_* is the Ohmic resistance of the electrolyte, *l* is the distance between the two SS electrodes, and *S* is the area of the electrodes.

The electrochemical window was determined using linear sweep voltammetry (LSV) experiment at a sweep rate of 1 mV s^−1^ from 0 to 5 V. To investigate electrochemical stability of electrolyte, symmetric Li||Li cells were assembled and cycled at current of 0.5 and 1 mA cm^−2^ with constant capacity of 0.5 and 1 mAh cm^−2^ for 400 hrs. The Li^+^ transference number (*t_Li_^+^*) of electrolytes was tested using the method proposed by Abraham *et al*. VMP−300 workstation was used to test the currents (including the initial (*I_0_*) and steady state (*I_ss_*)) of symmetric Li||Li testing cell under a small polarization potential (10mV). In the meantime, the initial and steady state values of the resistances (*R_0_* and *R_ss_*) were measured by EIS in initial stage and after steady state. The *t_Li+_* was then calculated according to the following equation

$t_{Li+}=\left( \frac{I_{ss}}{I_{0}} \right)*(\frac{R_{ss}(\Delta V-I_{0}R_{0})}{R_{0}(\Delta V-I_{ss}R_{ss})})$ Equation (2)

To evaluate the electrochemical performances of electrolytes, the CR2032 coin−type batteries were assembled using cathode of ferrous lithium phosphate (LiFePO_4_), ternary cathode (NCM811) and sulfurized polyacrylonitrile (SPAN), thin lithium anode, polyolefin separator, and electrolyte. All of cells were assembled in an argon−filled glovebox with O_2_ and H_2_O less than 0.1 ppm. The mass loading of active materials in cathode was 2 mg cm^−2^, and the thickness of lithium anode is 50 μm. The galvanostatic charge/discharge profiles of the LMB were measured by instrument of Land−2001A battery test system at 25 ℃

The assembling process of 1Ah LiFePO_4_||LB-002||Li pouch cells were assembled using LiFePO_4_ (18 mg cm^-2^) (60 * 80 mm^2^) as the cathodes and lithium foil (62 * 82 mm^2^) with a thickness of 50 μm as anodes, PP (Tianjin Kaipriet New Energy Technology Co., Ltd., China) as the separator, electrolyte with 1.5-1.8 g Ah^-1^ , and Al-plastic film (Changzhou Xinlun Composite Material Technology Co., Ltd., China) as packaging. All pouch cells are assembled using a “Z” stacking method. All preparations are conducted in a low-dew-point environment (< -60℃).

*1.4 Accelerating rate calorimeter (ARC) tests*

The ARC tests of 1Ah pouch cell were conducted with a BTC500 system purchased from HEL, England, equipped with a built-in digital camera and LAND test system. The “Heat-Wait-Search” model starting from room temperature with a heating step of 5 °C. The detected self-heating rate was 0.02 °C min^-1^. The temperature at that time is self-heating temperature (*T_1_*). Thermal runaway temperature (*T_2_*) was the temperature at which self-heat rate of cells reached 1 °C min^−1^.

2. Calculation methods

*2.1 MD simulations*

Molecular dynamic (MD) simulations were performed on the electrolyte mixtures (TMSA, FEC and LiTFSI) to observe the structure changes of the electrolyte mixtures. First, the optimized electrolyte molecules were packed in a periodic box to construct the bulk systems, the compositions of simulated electrolytes are given in **Table S1**. The molar ratios between the solvents and salts used in our simulations were 2: 1.67: 1 for TMSA: FEC: LiTFSI and 2: 1 for TMSA: LiTFSI. The simulation cells contained and 100 LiTFSI, 200 TMSA and 100 LiTFSI, 200 TMSA and 167 FEC, respectively. Subsequently, the energy minimization was used to remove potential overlaps among all molecules with the conjugate gradient minimizer method. Finally, all mixture systems were equilibrated by NPT (*i.e.*, isothermalisobaric) MD simulations for 5 ns at 298K and atmospheric pressure, followed by NVT (i.e., isothermal) MD simulations for 10 ns with a 1 fs time step. During the simulation, periodic boundary conditions were applied in all three dimensions. All MD simulations were performed using the Forcite code with COMPASSII force field.^[1]^ The temperature was controlled by a Nose-Hoover Langevin (NHL) thermostat and the pressure was controlled by a Berendsen barostat ^[2, 3]^. The Ewald scheme ^[4, 5]^ and atom-based cutoff method (*i.e.*, a radius of 15.5 Å) were applied to treat electrostatic and van der Waals (vdW) interactions, respectively. All the partial atomic charges were defined using the COMPASSII force field. The RDFs and coordinated number for electrolyte system was extracted to reveal the coordination structures between Li^+^ and TMSA, and FEC. The representative solvation structures from the MD simulations were also given.

*2.2 DFT calculations*

All quantum chemical calculations were performed by density functional theory (DFT) method using Gaussian 09 program package. The B3LYP functional was adopted for all calculations. For geometry optimization calculations, the 6-311+G(d, p) basis set was used, and the optimal geometry for each compound was determined.

**II. Supporting Tables**

Table S1. Molecular dynamic (MD) simulations of as-prepared electrolyte mixtures

|  | DES | DFM |
| --- | --- | --- |
| Number of TMSA per box | 200 | 200 |
| Number of FEC per box | / | 167 |
| Number of LiTFSI per box | 100 | 100 |
| Total number of atoms | 5800 | 7470 |
| Simulation box size (Å^3^) | 46.1×46.1×46.1 | 50.3×50.3×50.3 |
| MD, density (g/cm^3^) | 1.497 | 1.509 |
| Bias temperature (K) | 298 | 298 |

**III. Supporting Figures**


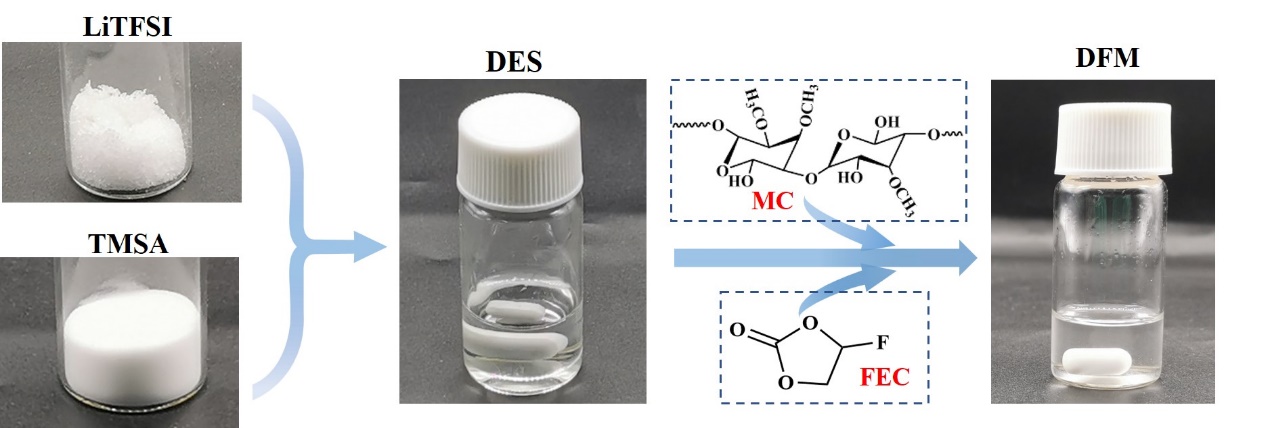


**Figure S1.** Schematic illustration of the electrolyte preparation.

Notes: The deep-eutectic-polymer electrolyte of DFM is prepared through introducing FEC and MC into DES. Specifically, TMSA blends with lithium salt of LiTFSI with molar ratio of 2:1 at room temperature to form DES, and then FEC and MC are added under stirring to obtain DFM.


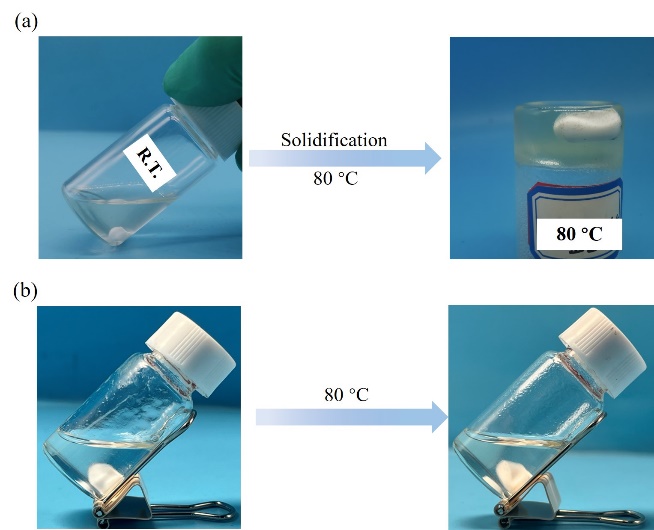


**Figure S2.** Digital images of (a) DFM electrolyte and (b) LB-002 electrolyte with MC at room temperature and 80 °C.

Notes: At the room temperature, MC disperses into solvent as isolated chains, thereby DFM keeps liquid state. When the temperature rises to 80 °C, MC chains automatically assemble to form dense gel networks quickly due to hydrophobic association, leading to electrolyte solidification. Although MC can dissolve in the LB-002 electrolyte, it cannot solidify upon heating, thereby the LB-002 with MC provides no benefit to enhancing battery safety.


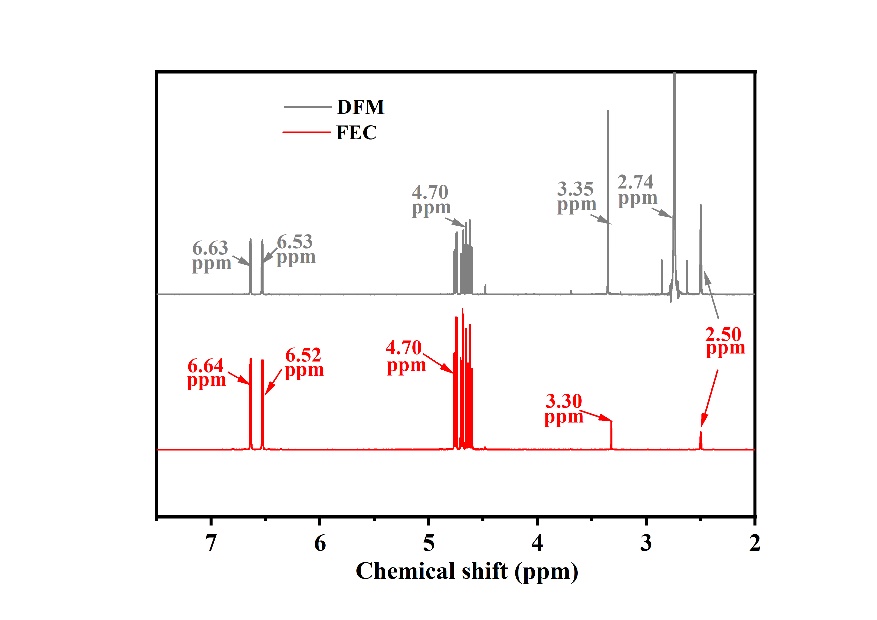


**Figure S3.** ^1^H nuclear magnetic resonance (^1^H NMR) spectra of the FEC and DFM.

Notes: As shown in Figure S3, the multiplets at 4.70 ppm correspond to -CH_2_- of FEC, and the peaks at 6.52 and 6.64 ppm are assigned to -CHF-. All FEC signals show negligible chemical shift in DFM, indicating that chemical environment of H (FEC) shows negligible change.


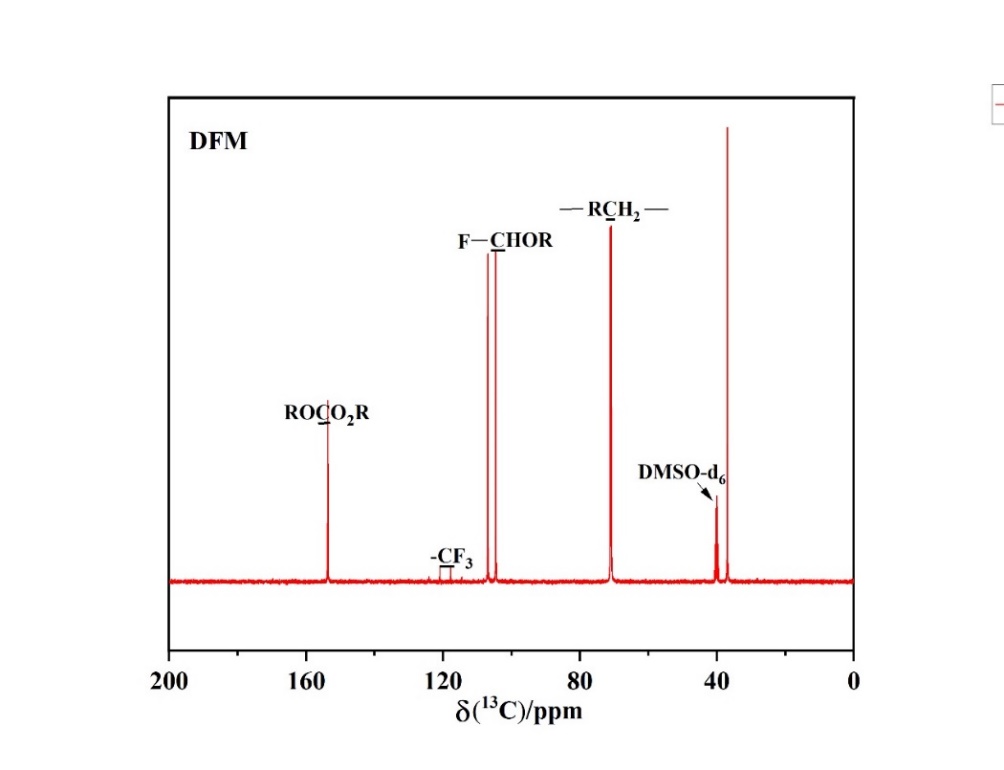


**Figure S4.** ^13^C nuclear magnetic resonance (^13^C NMR) spectra of the DFM.

Notes: The ^13^C NMR spectra of DFM was further conducted to analyze chemical environment of C atoms. The resonances C at 153.7, 104.6, and 70.1 ppm are assigned to FEC substitutes of ROCO_2_R, F-CHOR, and -RCH_2_R, respectively. ^[6, 7]^ The resonances at 120 and 37 ppm correspond to C in the LiTFSI and TMSA, respectively.^[8]^


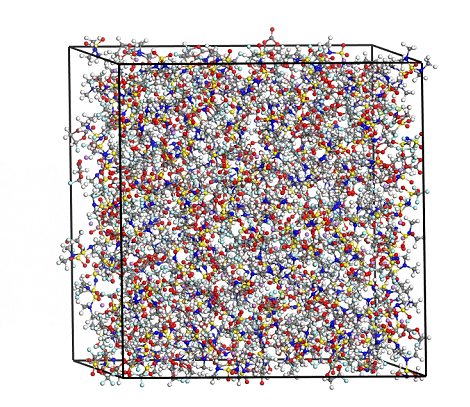


**Figure S5.** Molecular dynamics (MD) simulation snapshots of DFM obtained at 298 K.

Notes: Molecular dynamic (MD) simulations were performed on the electrolyte mixtures to observe the structure changes of the electrolyte mixtures. The optimized electrolyte molecules were packed in a periodic box to construct the bulk systems.


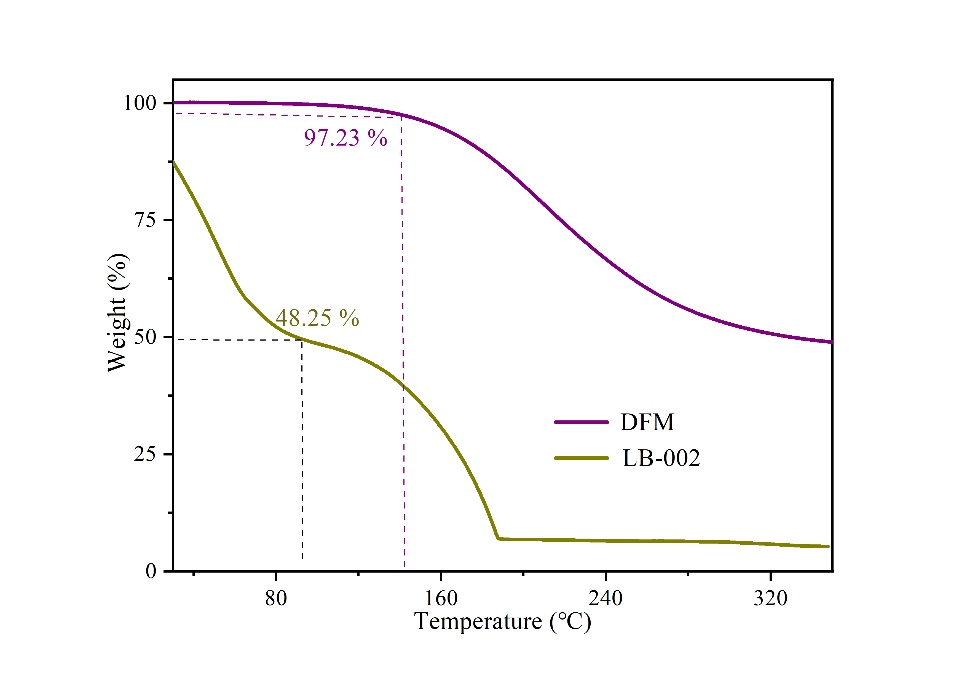


**Figure S6.** Thermal gravimetric curves of DFM and LB-002 electrolytes.

Notes: DFM displays a low weight loss of 2.77% until 150 °C due to its low vapor pressure, suggesting that neither volatilization nor degradation takes place in the DFM electrolyte. As comparison, the commercially available electrolyte (LB-002) loses 4.79% of original weight at room temperature, and presents a weight loss of 51.77% at 150 °C.


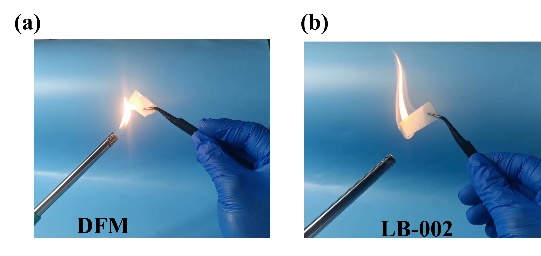


**Figure S7.** Digital images of (b) DFM electrolyte and (c) LB-002 under combustion test.

Notes: The flammability of DFM is investigated through combustion test. As shown in Figure S7, the DFM cannot be ignited, while LB-002 burns violently when it is close to flame. The high thermal stability and excellent nonflammability of DFM would drastically improve the safe property of high energy density LMBs.


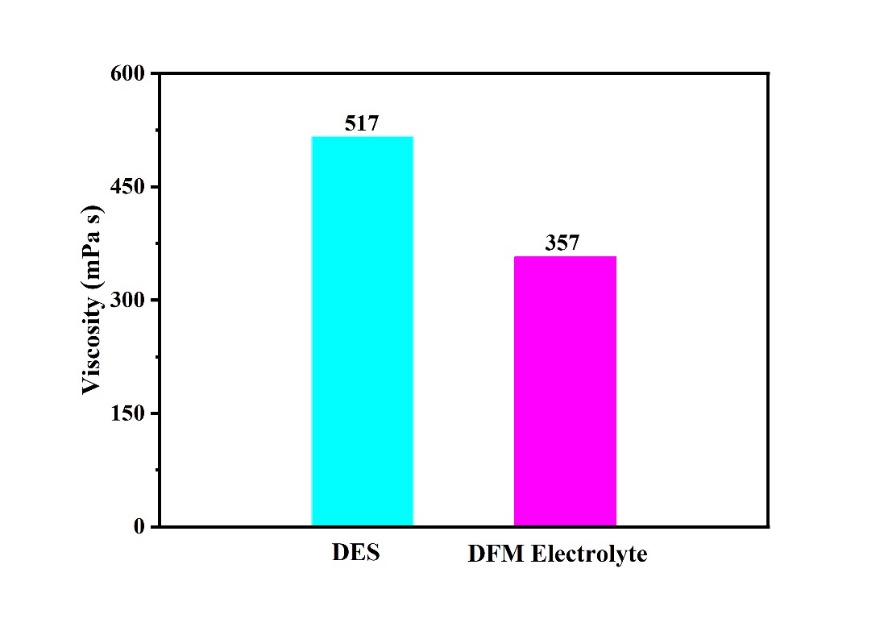


**Figure S8.** The viscosity data of prepared electrolyte.

Notes: The viscosity of DES is 517 mPa s, while that of DFM is 357 mPa s, indicating that the addition of FEC decreases electrolyte viscosity. The low viscosity of DFM facilitates Li^+^ transport in electrolyte, resulting in improvement on ionic conductivity.


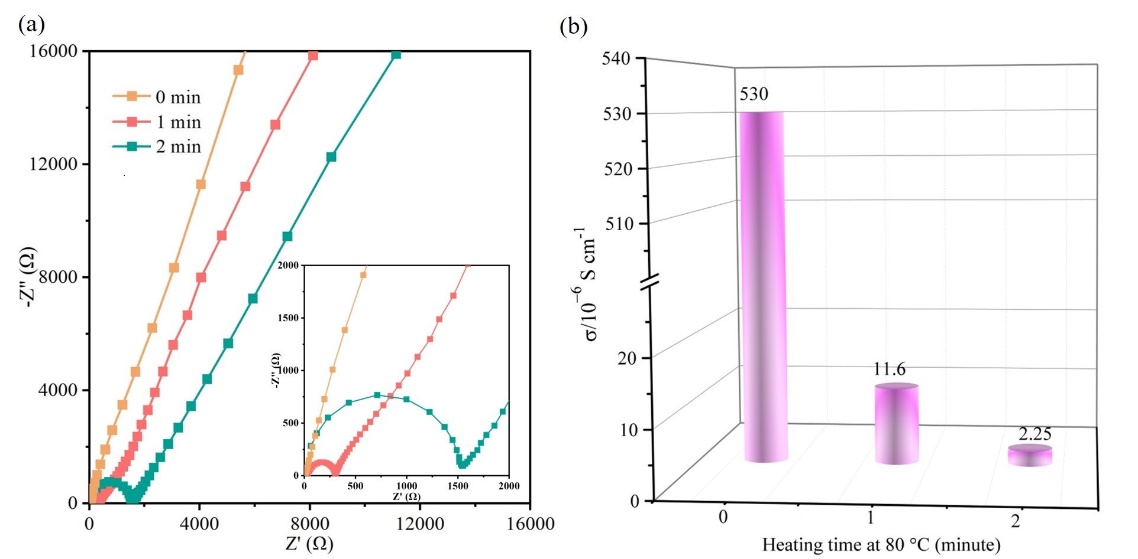


**Figure S9.** (a) Electrochemical impedance spectroscopy (EIS) spectra of SS||DFM||SS cells and (b) Ionic conductivity of DFM electrolyte after undergoing heat treatment for 0, 1 and 2 mins at 80 °C.

Notes: In order to estimate ionic conductivity of DFM electrolyte after different heating time at 80 °C, a series electrochemical impedance spectroscopy measurements based on SS||DFM||SS cells were conducted. As shown in Figure S9, the ionic conductivity of DFM at room temperature is 5.30 × 10^-4^ S cm^-1^, while it dramatically reduces along with increase of heating time at 80 °C. The ionic conductivity of DFM after heating at 80 °C for 1 and 2 minutes is 1.16 × 10^-5^ and 2.25 × 10^-6^ S cm^-1^, respectively.


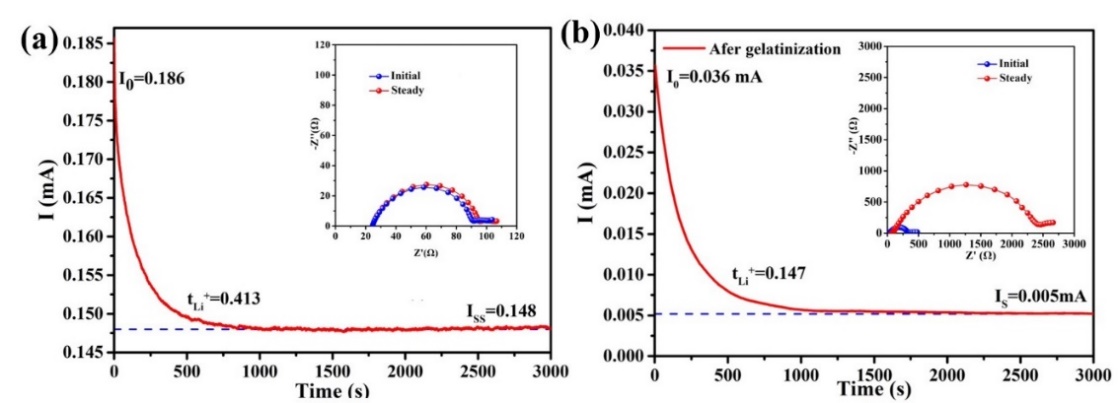


**Figure S10.** The chronoamperometry profiles of Li||DFM||Li cells at room temperature (a) and after gelation at 80 °C (b) under a polarization voltage of 10 mV. The corresponding electrochemical impedance spectroscopy (EIS) spectra before and after stabilization are shown in the inset.

Notes: DFM shows a Li^+^ transference number (*t_Li+_*) of 0.43 at room temperature. After solidification, Li^+^ ions are trapped in polymer networks due to relatively strong interaction between Li and functional substitute of MC, thereby the *t_Li+_* of DFM dramatically decreases to 0.147. The obvious reduce of ionic conductivity and *t_Li+_* under thermal stimulation is the basis of overheating self-protection of DFM.


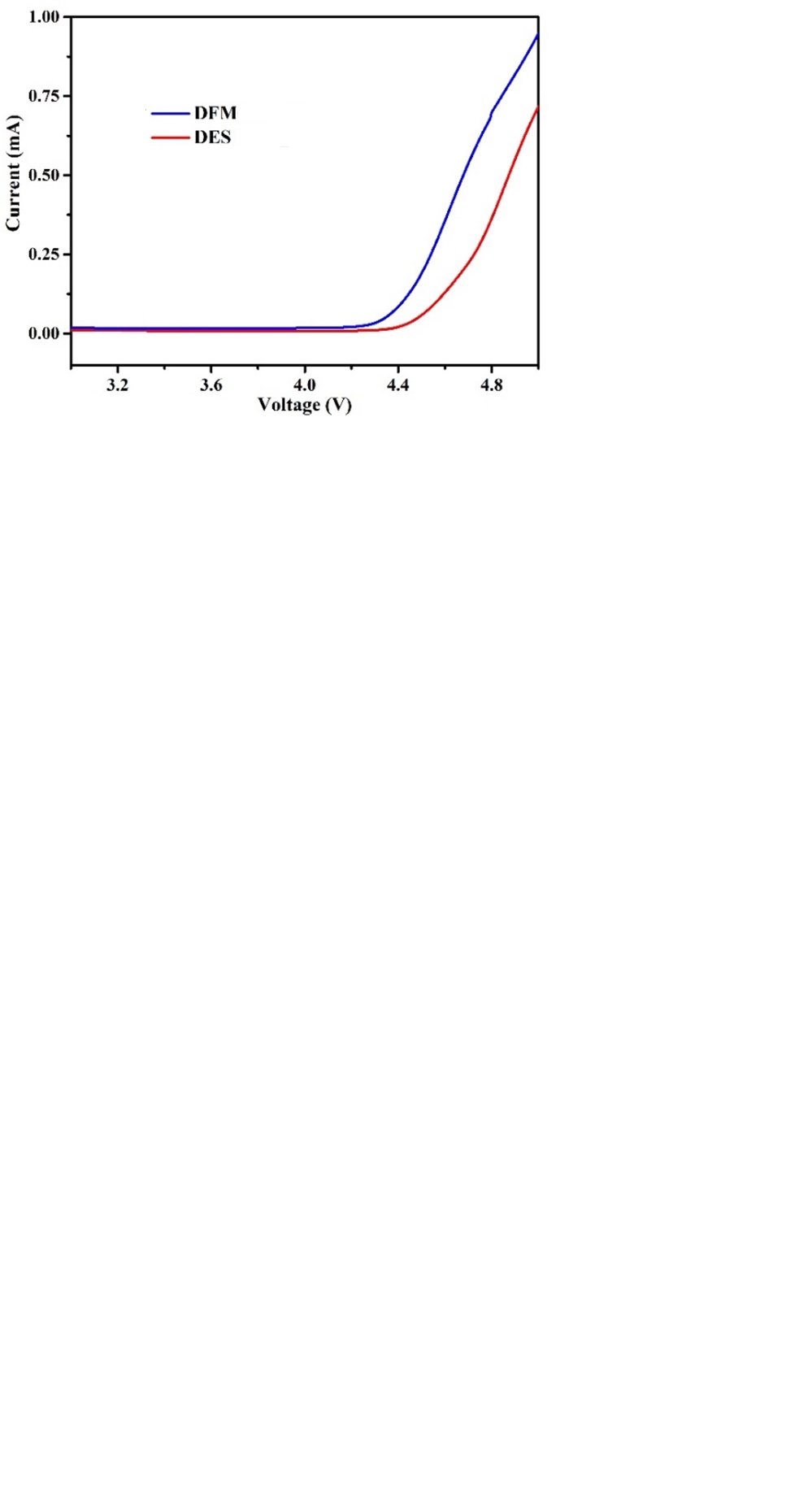


**Figure S11.** Linear sweep voltammetry **(**LSV) of the DES and DFM electrolytes at a scan rate of 5 mV s^-1^ using stainless steel as working electrode, and Li as counter and reference electrodes.

Notes: Liner-sweep voltammetry (LSV) was conducted to evaluate electrochemical stability of electrolytes. The oxidation current of DES is lower than 20 µA until 4.40 V, while DFM electrolyte is oxidized at voltage above 4.32 V. The reason for lower stable potential of DFM may be that FEC easily decomposes at electrode surface.


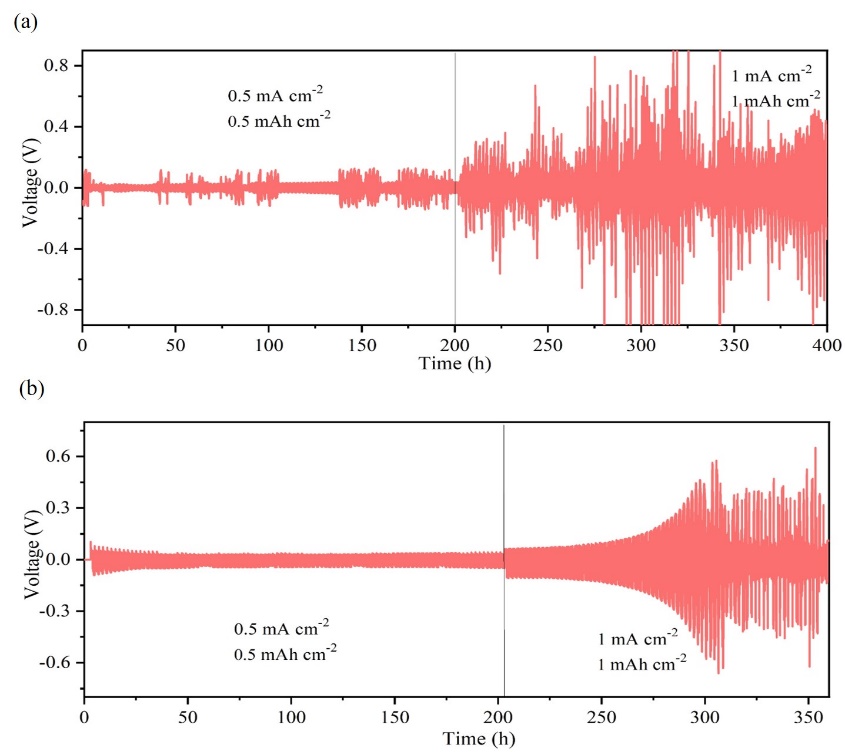


**Figure S12.** Voltage profiles of Li||Li symmetric cells employing (a) DES electrolyte and (b) commercially available electrolyte of LB-002 at 0.5 and 1 mA cm^-2^ with a capacity of 0.5 and 1 mAh cm^-2^, respectively.

Notes: As shown in Figure S12, the Li||DES||Li cell shows asymmetric plating/striping voltage files with overpotential of 0.1 V at 0.5 mAh cm^-1^, indicating unstable Li/electrolyte interfaces. Additionally, dramatically increased overpotential is observed when current density rises to 1 mAh cm^-2^. The results suggest that parasitic reaction between DES and Li metal takes place continuously because DES cannot form protective SEI on Li surface. The Li||LB-002||Li cell displays a stable overpotential of 66 mV at current density of 0.5 mA cm^-2^. However, when the current density increases to 1 mAh cm^-2^, the overpotential of cells distinctly increases to 0.5 V due to inferior SEI


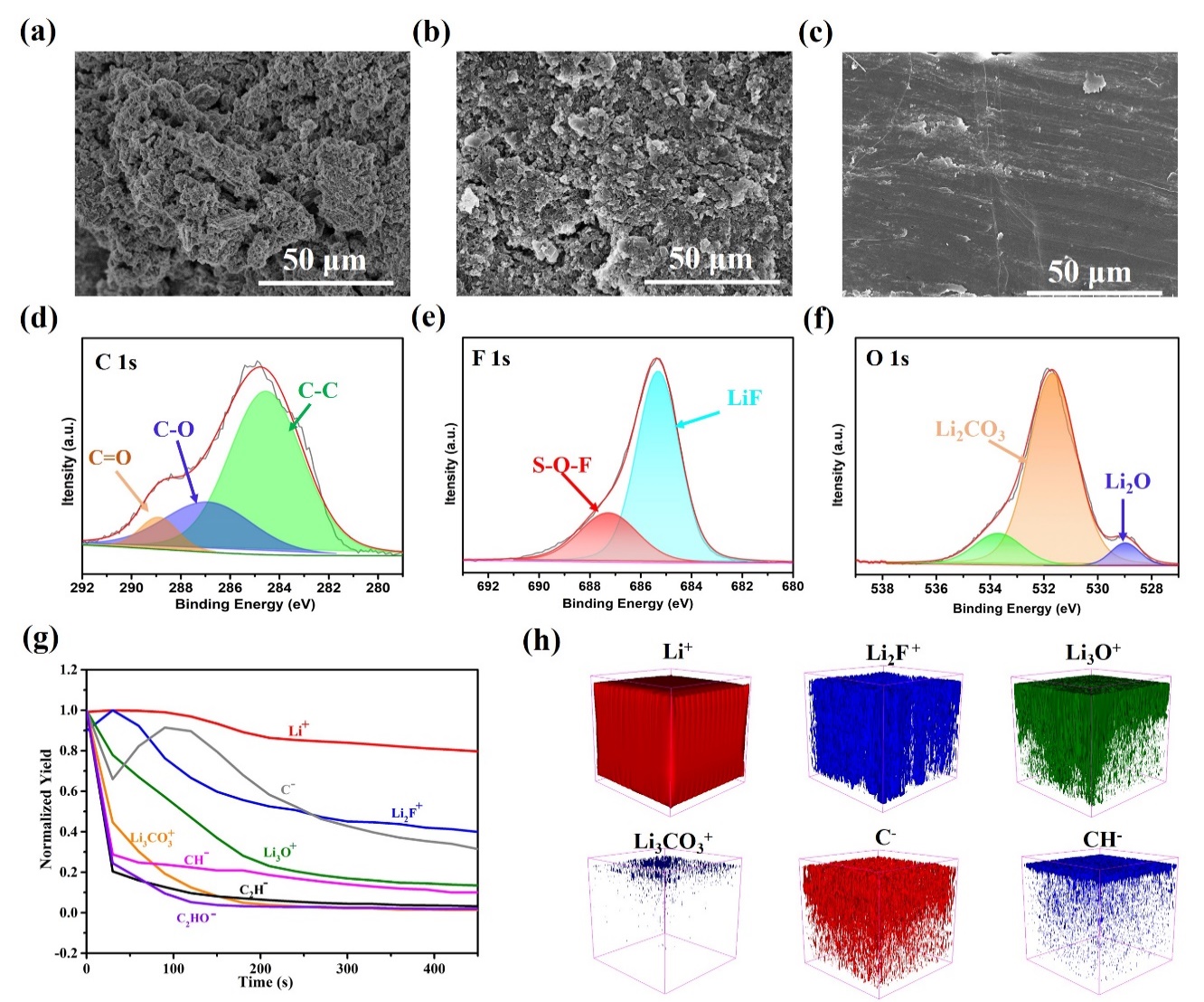


**Figure S13.** SEM image of Li electrodes after Li plating/stripping 200 hours in (a) Li||LB-002||Li and (b) Li||DES||Li cells.

Notes: The Li anode obtained from cycled Li||LB-002||Li cell displays an obviously porous and loose surface due to uneven Li disposition and parasitic interface reaction. Similarly, apparent particles and cracks can be found on surface of Li electrode harvesting from cycled Li||DES||Li cell, verifies that DES hardly achieve homogeneous Li deposition.


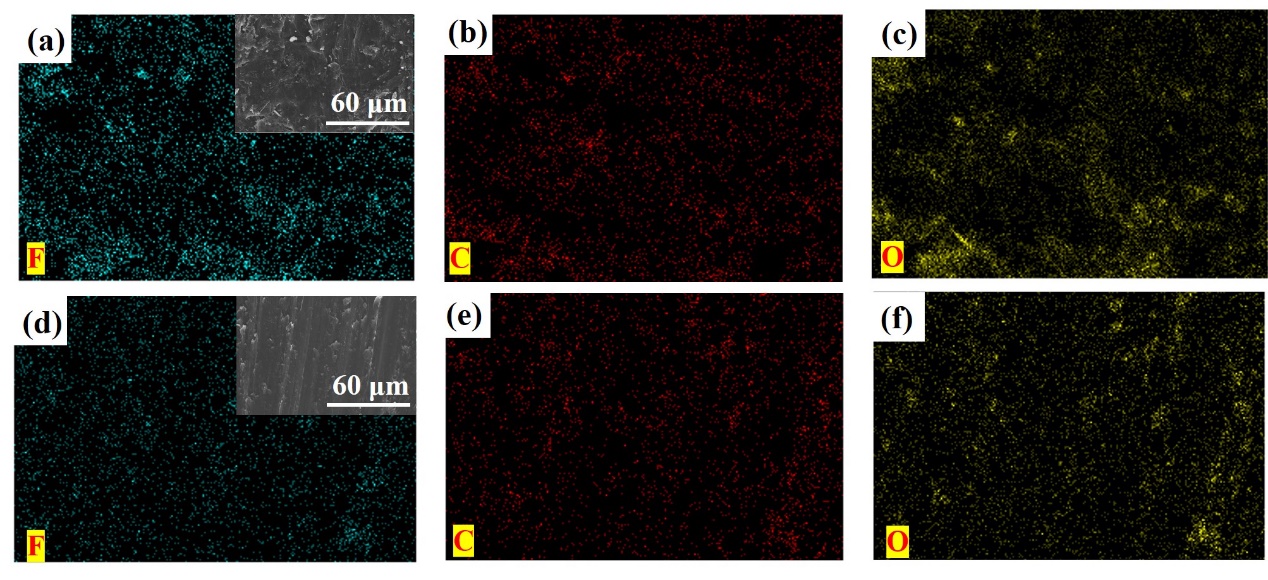


**Figure S14.** Elemental mapping of Li electrodes after plating/stripping 200 hours in symmetric cells of (a, b, c) Li||DES||Li and (d, e, f) Li||DFM||Li. The insets are the selected areas for mapping.

Notes: The energy-dispersive X−ray spectrometer (EDS) was employed to evaluate the elemental distribution on surface of cycled Li. F is found on Li of Li||DES||Li, which confirm that the existence of F-containing species in SEI deriving from decomposition of Li salt. Compared with Li||DES||Li, Li||DFM||Li shows more homogeneous distribution of F, C and O, indicating that DFM facilitate formation of uniform SEI.


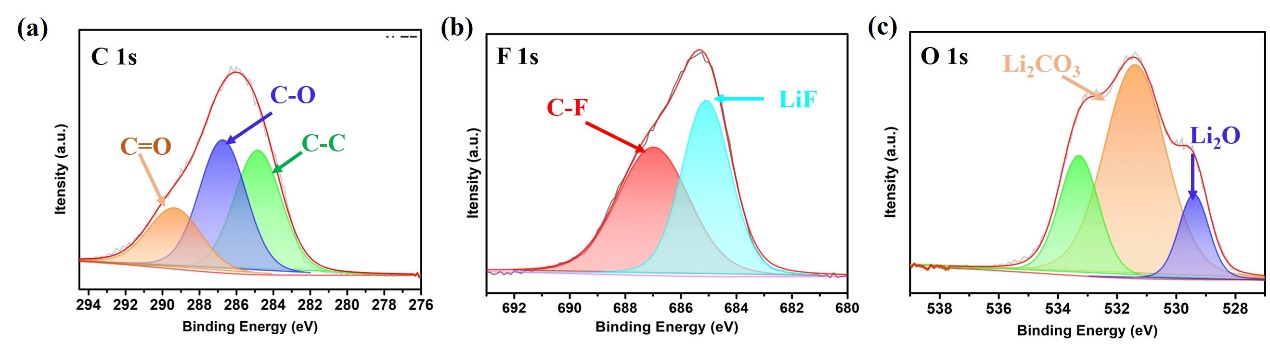


**Figure S15.** XPS spectra (C1s, O1s and F1s) of Li electrodes cycled in Li||DES||Li symmetric cells with DES electrolytes (d, e, f).

Notes: The characteristic signals for C-C, C-O and C=O is observed at 284.6, 286.8 and 289.8 eV in C 1s spectra of Li||DES||Li symmetric cells, verifying the existence of organic species, LiCOOR, and Li_2_CO_3_. In addition, the signals for LiF and Li_2_CO_3_ are found in Li1s and C1s spectra, respectively. Compared with SEI formed in Li||DFM||Li cells, that in Li||DES||Li displays lower content of inorganic LiF and Li_2_CO_3_.


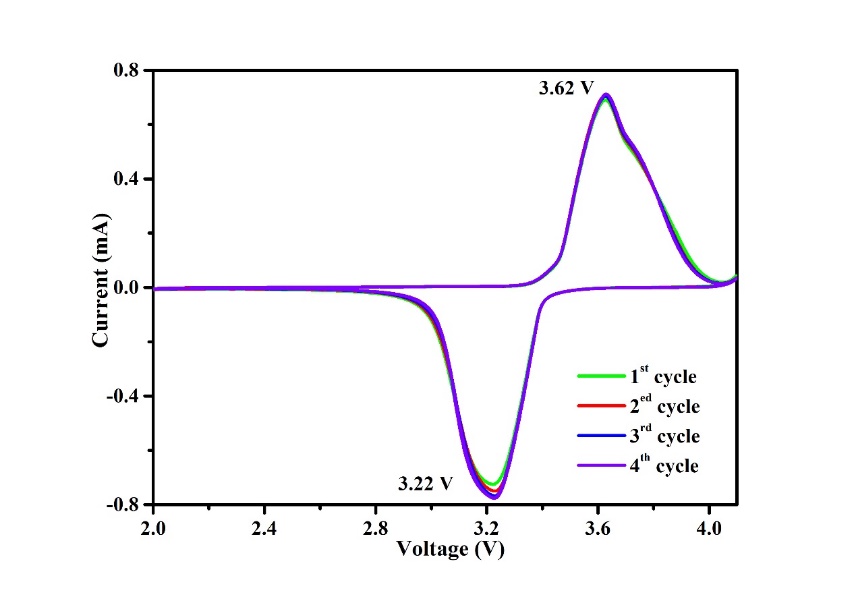


**Figure S16.** Cyclic voltammograms of LiFePO_4_||DFM||Li at 0.5 mV s^−1^.

Notes: CV curves of LiFePO_4_||DFM||Li display a pair of peaks at 3.2 and 3.6 V, corresponding to typical reduction and oxidation processes of LiFePO_4_. Additionally, overlap of the CV curves demonstrate that electrodes reactions show excellent reversibility.


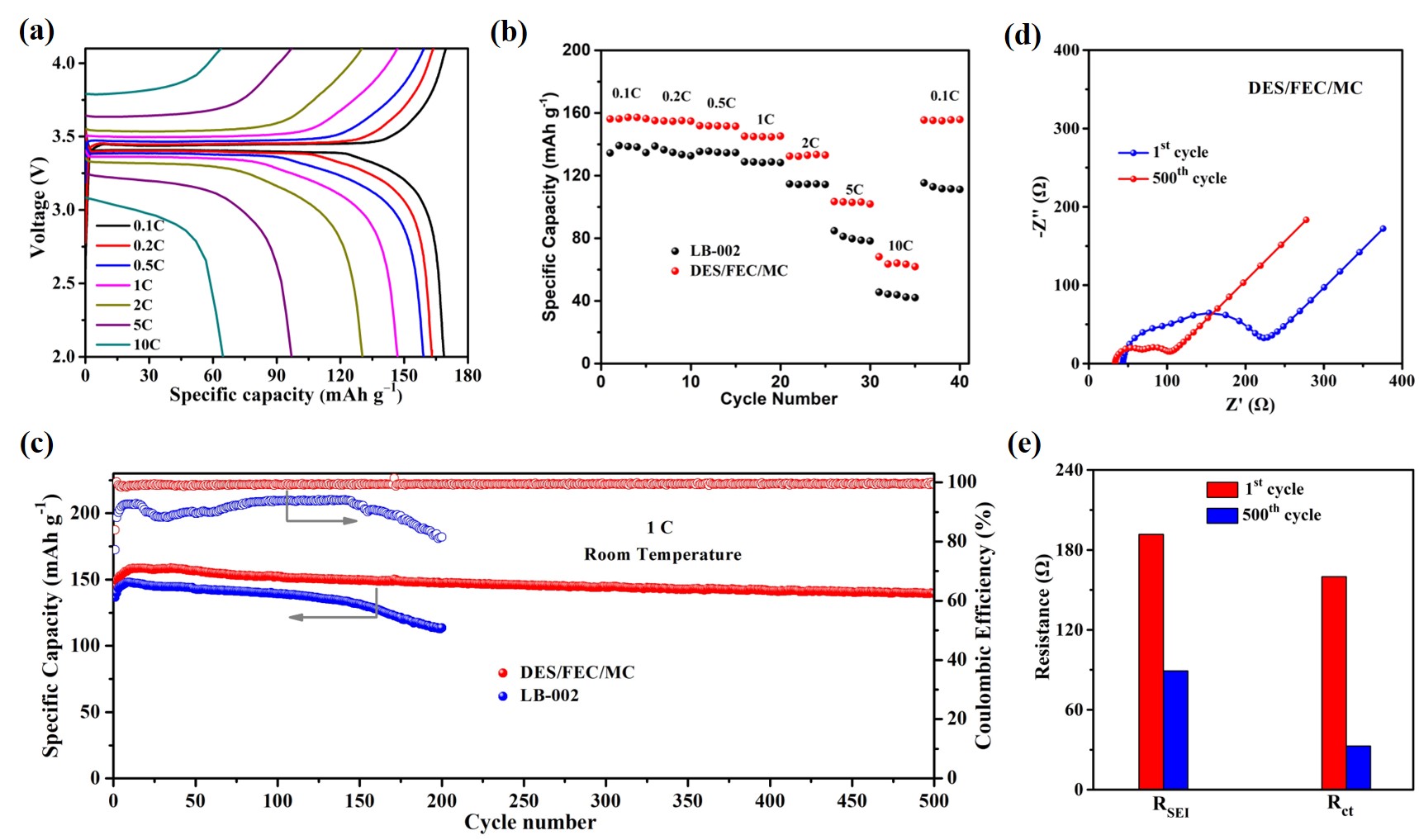


**Figure S17.** Cycle performance of LiFePO_4_-based LMBs with DFM and LB-002 at different current density.

Notes: The initial discharge capacity of LiFePO_4_||DFM||Li batteries is 167 mAh g^−1^ at 0.1 C, which is close to theoretically specific capacity of LiFePO_4_. With the raise of current density, the polarizations of voltage profiles increase owing to limitation of reaction kinetics, reducing discharge capacity to some extends. The LiFePO_4_||DFM||Li battery delivers specific capacities of 162, 158, 146, 130, 97, 65 mAh g^−1^ at 0.2, 0.5, 1, 2, 5 and 10 C, respectively. As comparison, the LiFePO_4_||LB-002||Li battery delivers specific capacities of 140, 136, 135, 130, 115, 78, 43 mAh g^−1^ at 0.1, 0.2, 0.5, 1, 2, 5 and 10 C.


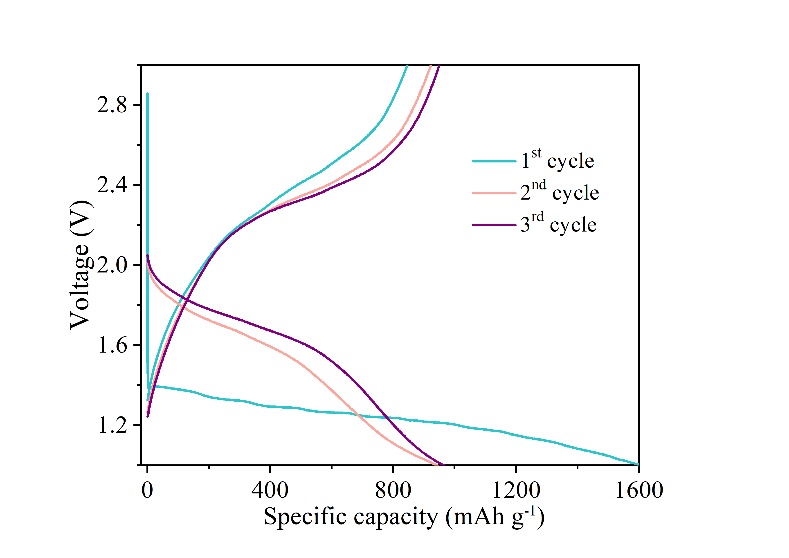


**Figure S18.** Galvanostatic charge/discharge profiles of sulfurized polyacrylonitrile based LMBs (SPAN||Li) DFM at 0.5 C in voltage of 1-3 V.

Notes: Figure S18 shows galvanostatic charge/discharge profiles of SPAN||DFM||Li battery. In the first discharge process, the voltage plateau potential of 1.3 V corresponds to irreversible conversion of original SPAN with unique Li^+^ storage mechanism in its double bonds. In subsequent cycles, reversible rupture and re-formation of S−S bonds in SPAN are achieve, giving reversible discharge capacity of 945 mAh g^-1^.


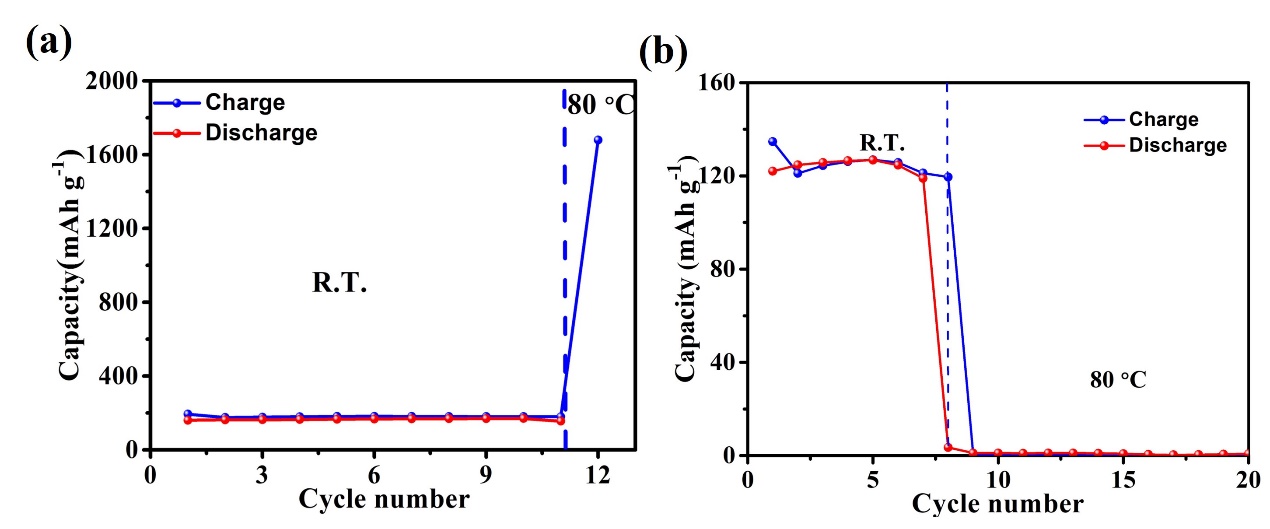


**Figure S19.** Cycle performances of LiFePO_4_||LB-002||Li batteries at room temperature and 80 °C with current density of 1 C.

Notes: LiFePO_4_||LB-002||Li displays stable cycle performance with a specific capacity of 150 mAh g^-1^ at room temperature and 1 C. However, when the temperature increases to 80 °C, serious overcharge is observed in LiFePO_4_||LB-002||Li with specific capacity of 1600 mAh g^-1^ due to the occurrence of internal short-circuit, which increases risk of thermal runaway. In contrast, the LiFePO_4_||DFM||Li battery deliver no capacity at elevated temperature of 80 °C. The results confirm the shut-down function of the DFM electrolyte.


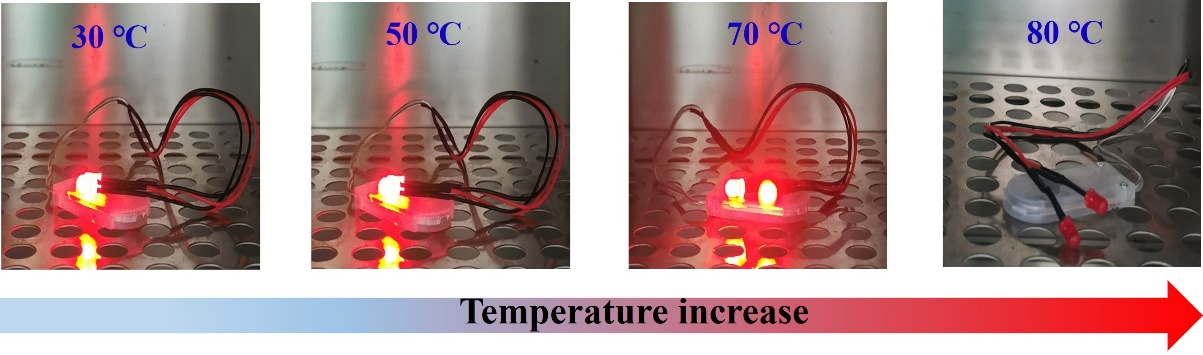


**Figure S20.** Images exhibition of luminous diode powered by LiFePO_4_||DFM||Li battery at 30, 50, 70 and 80 °C.

Notes: The luminous diode emits red light when it is connected to the LiFePO_4_||DFM||Li battery at 30, 50, 70°C. However, the solidification of the as-prepared electrolyte terminates charge/discharge process of the batteries, resulting in that the luminous diode goes off at 80°C.


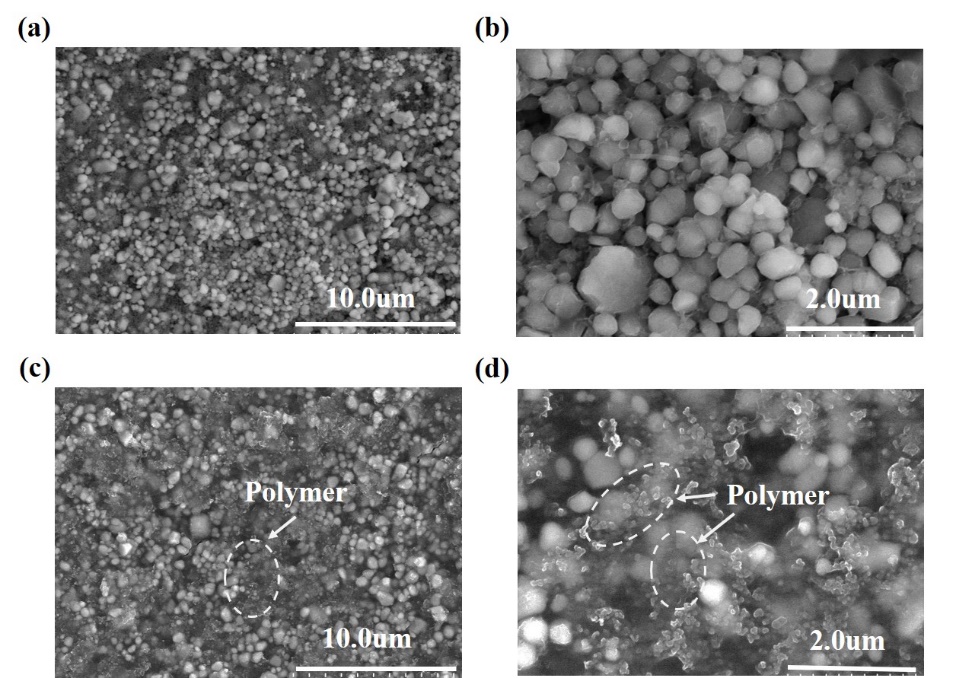


**Figure S21.** SEM images of LiFePO_4_||DFM||Li cathode at room temperature (a, b) and 80 °C (c, d).

Notes: Figure S21 shows morphologies of LiFePO_4_||DFM||Li cathode at room temperature (a) and 80 °C (b). At room temperature, DFM electrolyte is liquid, thereby there is no polymer on cathode surface. However, when DFM solidifies at 80 °C, a layer of polymer covers most part of cathode surface. The polymer layer inhibits Li^+^ transport through electrolyte/cathode interfaces and increases interfacial resistance because of its low ionic-conductivity, thereby the electrolyte reactions are terminated at high temperature.


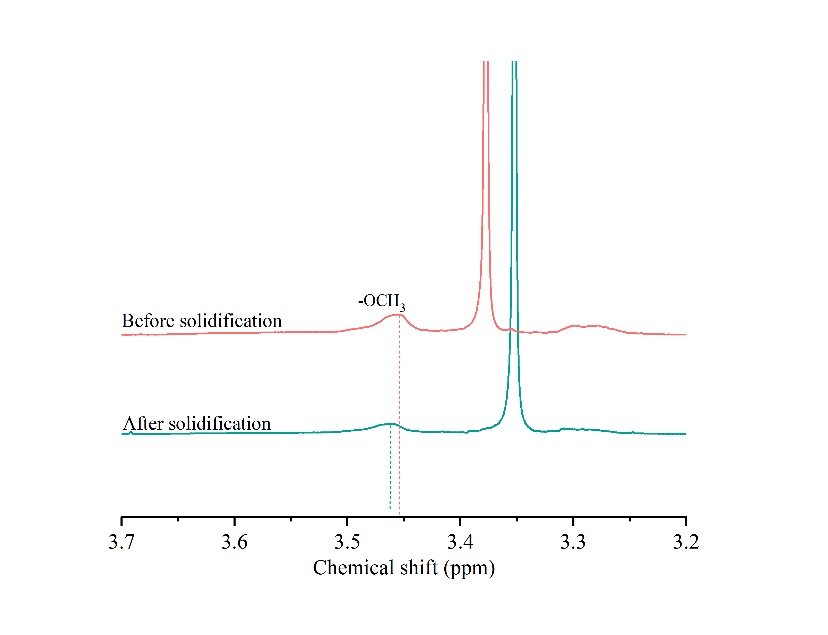


**Figure S22.** ^1^H NMR spectra of DFM electrolyte before and after electrolyte solidification.

Notes: ^1^H NMR was conducted to evaluated the changes on intermolecular interactions between MC. The proton resonances of -OCH_3_ in MC decrease obviously after electrolyte solidification. The reason is that, at elevated temperature, the mobility of MC chains decreases due to the enhancement of hydrophobic interactions between –OCH_3_ of polymer chains. Additionally, it can be found that the proton resonance of -OCH_3_ shifts to lower resonance frequency after electrolyte solidification due to interactions between -OCH_3_.^[9]^


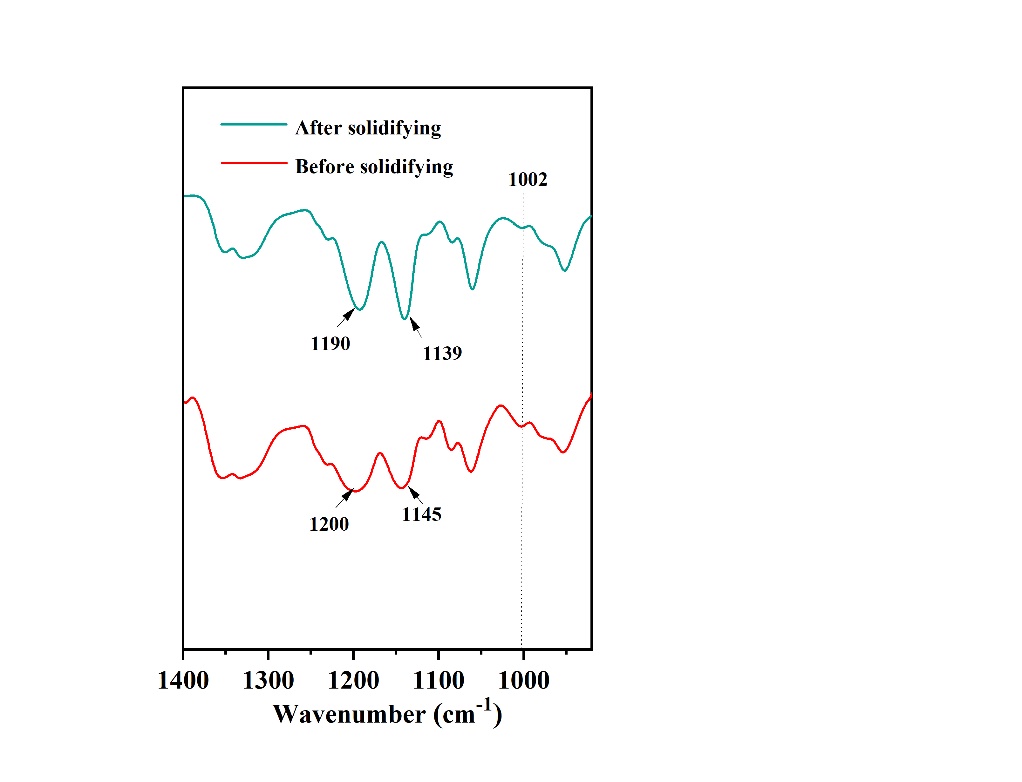


**Figure S23.** FTIR spectra of DFM electrolyte before and after solidifying.

Notes: The FTIR spectra provide further information on molecular interactions. On the spectra of DFM before solidifying, the peaks at 1200, 1100 and 1080 cm^-1^ are assigned to stretching of C-O (ν_C-O_).^[10]^ After electrolyte solidification, the peaks at 1200 and 1145 cm^-1^ display red shift of 10 and 6 cm^-1^, suggesting that the interaction between -OCH_3_ groups are enhanced due to hydrophobic association of MC.

**References**

[1] H. Sun, *J. Phys. Chem. B* **1998**, *102*, 7338–7364.

[2] A. A. Samoletov, C. P. Dettmann, M. A. Chaplain,*J. Stat. Phys.* **2007**, *128*, 1321–1336.

[3] H. J. Berendsen, J. V. Postma, W. F. van Gunsteren, A. DiNola, J. R. Haak, *J. Chem. Phys.* **1984**, *81*, 3684–3690.

[4] P. P. Ewald, *Ann. Phys.* **1921**, *369*, 253–287.

[5] M. P. Tosi, *Solid State Phys.* **1964**, *16*, 1–120.

[6] Q. Sun, S. Wang, Y. Ma, D. Song, H. Zhang, X. Shi, N. Zhang, L. Zhang, *Adv. Mater.* **2023**, *35*, 2300998

[7] W. Li, L. Ma, S. Liu, X. Li, J. Gao, S. Hao, W. Zhou, *Angew. Chem. Int. Ed.* **2022**, *61*, e202209169

[8] Y. Jin, N.H. Kneusels, L.E. Marbella, E. Castillo-Martinez, P.C.M.M. Magusin, R.S. Weatherup, E. Jonsson, Tao. Liu, S. Paul, C.P. Grey, *J. Am. Chem. Soc.* **2018**, 140, 9854−9867

[9] Y. Ding, Y. Yan, Q. Peng, B. Wang, Z. Hua, Z. Wang, *ACS Appl. Polym. Mater.* **2020**, *2*, 3259−3266

[10] L. Li, P.M. Thangamathesvaran, *Langmuir* **2001**, *17*, 8062-8068
